# A multi-omics approach reveals specific oncogenic and inflammatory potential of viral oncoproteins Tax from neglected HTLV-1b and -1c genotypes

**DOI:** 10.1371/journal.ppat.1014512

**Published:** 2026-08-25

**Authors:** Thomas Duchateau, Alice Bongers, Karim Abdelmoumen, Carine Rey, Sébastien Dussurgey, Jacques Brocard, Dalia Gerardin, Marie-Lou Béziat, Florence Lormières, Devashish Bhat, Christophe Guillon, Patrice Gouet, Philippe V. Afonso, Chloé Journo

**Affiliations:** 1 Centre International de Recherche en Infectiologie, Retroviral Oncogenesis Team, Inserm U1111, Université Claude Bernard Lyon 1, CNRS UMR5308, École Normale Supérieure de Lyon, Université Lyon, Lyon, France; 2 Unité Épidémiologie et Physiopathologie des Virus Oncogènes, Institut Pasteur, Université Paris Cité, Paris, France; 3 Centre International de Recherche en Infectiologie, BioInformatic and BioStatistics Team, Inserm U1111, Université Claude Bernard Lyon 1, CNRS UMR5308, École Normale Supérieure de Lyon, Université Lyon, Lyon, France; 4 Universite Claude Bernard Lyon 1, CNRS UAR3444, Inserm US8, ENS de Lyon, SFR Biosciences, Lyon, France; 5 Indian Institute of Science Education and Research, Tirupati, India; 6 Molecular Microbiology and Structural Biochemistry, Retroviruses and Structural Biochemistry Team, CNRS UMR5086, Université Claude Bernard Lyon 1, Lyon, France; Penn State Health Milton S Hershey Medical Center, UNITED STATES OF AMERICA

## Abstract

Over the recent years, reports of very high prevalence of Human T-cell Leukemia Virus (HTLV) type 1 genotype c infection in remote aboriginal populations in Central Australia have sparked a renewed interest in the genetic variability of this human oncogenic retrovirus. While clinical and epidemiological studies suggest that HTLV-1 genotypes might be associated with varying risks of pathological manifestations, molecular comparisons of the viral determinants among genotypes remain scarce. In this study, we provide the first comprehensive comparative analysis of the major oncoprotein Tax1 from genotypes a (Japanese), b (African) and c (Australo-Melanesia). Using unbiased analysis of image cytometry data from Tax1-expressing Jurkat T-cells, combined with proximity labelling, we first show that Tax1 variants exhibit distinct subcellular localization in T-cells. Indeed, in contrast to Tax1a that assembles a NF-κB-activating signalosome associated with the Golgi apparatus, Tax1c variants lack any significant association with this cell compartment. Surprisingly however, Tax1c does not show any general defect in NF-κB activation compared to Tax1a and Tax1b. Nevertheless, transcriptomics analysis combined with pathway inference indicate that the quality of the NF-κB signature induced by Tax1c differs from that of Tax1a, and that the transcriptional landscape of Tax1c-expressing cells is biased towards T-cell activation and inflammation, an observation that is consistent with the kinome profiling of Tax1c-expressing cells. Analysis of Tax1b-induced transcriptional reprogramming revealed that it closely parallels that of Tax1a, but with a higher magnitude. In addition, distinct modulation of cell cycle-related kinase activity by Tax1b compared to Tax1a was correlated with a specific modulation of the cell cycle. Consistently, functional cell transformation assays demonstrated that Tax1b presents a higher oncogenic potential compared to Tax1a, while Tax1c harbours a decreased transforming activity. Altogether, we provide evidence of functional divergence among Tax1 from different genotypes that may have pathophysiological and clinical implications.

## Author summary

The Human T-cell Leukemia Virus type 1 (HTLV-1) is the only cancer-causing retrovirus described in humans. It infects at least 10 million individuals throughout the world, with regions of high prevalence in Japan, Latin and South America, Central Africa and Australo-Melanesia. HTLV-1 strains are subdivided into distinct groups that reflect their geographical origin, with HTLV-1b circulating in Central Africa and HTLV-1c in Australia. Epidemiological data suggest stark geographical differences in the incidence of HTLV-1-associated diseases. However, most molecular studies have focused on the cosmopolitan HTLV-1a strains. Here, we provide the first comprehensive comparative analysis of the major oncogenic protein Tax1 from HTLV-1a (cosmopolitan), -b (African) and -c (Australo-Melanesia) strains. We show that while Tax1a and Tax1b could favour oncogenic pathways in cells, Tax1c could rather favour inflammatory pathways, consistent with clinical and epidemiological observations. Taken together, our results call for further investigations on neglected or minor HTLV-1 strains.

## Introduction

The Human T-cell Leukemia Virus type 1 (HTLV-1) is the only oncogenic retrovirus described in humans [1]. While more than 80% of the estimated 5–10 millions people living with HTLV-1 remain lifelong asymptomatic carriers, chronic infection has been associated with severe pathologies including Adult T-cell Leukemia/Lymphoma (ATL) [1] and HTLV-1-Associated Myelopathy/Tropical Spastic Paraparesis (HAM/TSP) [2], as well as with a spectrum of inflammatory diseases including infective dermatitis associated with HTLV-1 [3,4], bronchiectasis [5], uveitis and Sjogren’s syndrome [6]. Of note, the lifetime risk of developing HTLV-1-associated diseases varies between regions of the world (for a recent review, see [7]). For instance, HTLV-1-associated bronchiectasis is the major HTLV-1-associated disease in Australia [8], while this clinical manifestation is rare in Europe, the Americas and Japan. In the past decades, various host-related factors, including age at primary infection, transmission routes and host genetic background, have been shown to contribute to differences in HTLV-1 clinical outcomes. Whether viral phylogenetic diversity could also contribute to these clinical and epidemiological differences is currently unclear. Most of the current understanding of HTLV-1 molecular features stems from studies on the prototypic ATK strain (belonging to the HTLV-1a Japan subtype; formerly known as HTLV-1aB) and on HTLV-1a-TC (transcontinental) strains. While reports of strikingly high prevalence of infection with HTLV-1 genotype c in remote aboriginal populations in Central Australia [9] have prompted very recent studies on the molecular and immunological aspects of infection by this divergent genotype restricted to Australia and Melanesian archipelagos [10,11], much less is known on the molecular features of HTLV-1b, the most common variant in Central Africa (for reviews, see [12,13]).

Multiple studies on HTLV-1a, combining cellular and animal models, have demonstrated the key role of the viral transactivator Tax1a in the transcriptional reprogramming and oncogenic transformation of infected T-cells [10,14–18]*.* Tax impairs biological processes such as the cell cycle [19,20], apoptosis [21,22], p53-dependent response [23], and DNA repair [24], and modulates host cell signalling pathways including the NF-κB [25], CREB [26], and MAPK pathways [27]. Tax thus modulates the transcriptional program of host cells [28,29], but also post-transcriptional processes such as alternative splicing [30–32]. In this work, we hypothesized that the Tax1 proteins from distinct HTLV-1 genotypes induce specific reprogramming of host cells, and tested this hypothesis by providing a comprehensive comparative analysis of Tax1 variants in Jurkat T-cells using image cytometry, proximity labelling, transcriptomics and kinome profiling, combined with functional transformation assays.

## Results

### Selection of Tax1 variants representative of HTLV-1 phylogenetic diversity

**Fig 1:**
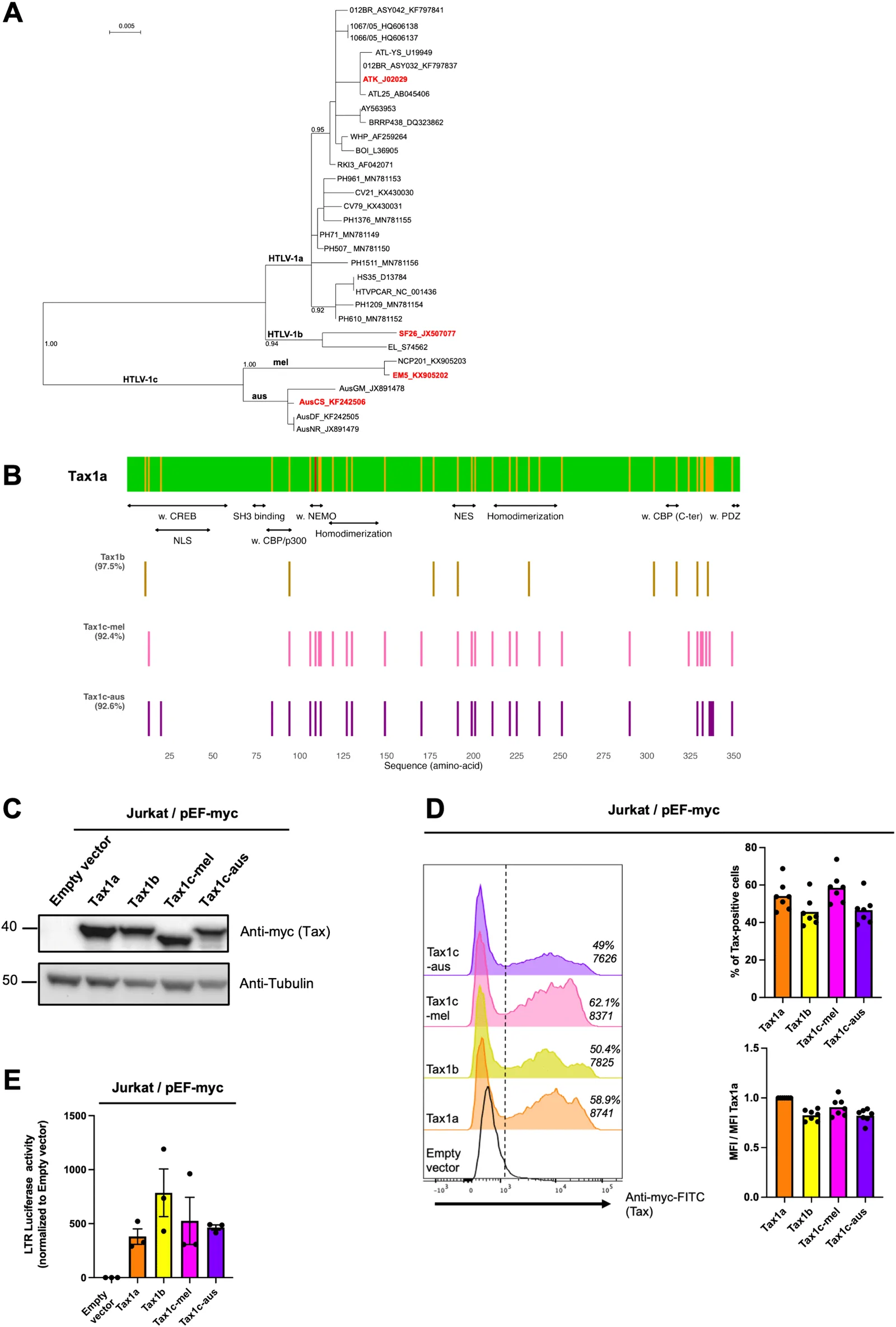
Selection of Tax1 variants representative of HTLV-1 phylogenetic diversity and validation of detection in Jurkat T-cells. **A.** The phylogenetic tree was constructed using the maximum likelihood method based on 26 *tax* sequences (984 nt). The branch lengths are drawn to scale with bar indicating 0.005-nucleotide substitution by site. The branch support values were estimated using the approximate likelihood-ratio test (aLRT). Genbank accession numbers are presented. The sequences used in this study are presented in red. **B.** Amino acid sequence alignment of the four Tax1 variants compared in the present study. Vertical bars represent variation in amino acid sequences in Tax1b, Tax1c-mel and/or Tax1c-aus compared to Tax1a. Double black arrows represent the main functional domains described in Tax1a. **C-D.** Jurkat T-cells were microporated with the pEF-Tax1-myc constructs and cultured for 20h before analysis by western blot **(C)** or flow cytometry **(D)** using anti-myc antibodies. **(C)** Tubulin was used as a loading control in western blot. **(D)** After gating on single live cells, the percentage of Tax-myc positive cells and the mean fluorescence intensity (MFI) were determined. The left panel shows a representative replicate (dotted line represents the Tax^+^ gate, and percentage of Tax^+^ cells and MFI of this replicate are indicated in italic), and values for independent replicates are shown on the right panel, where the MFI was normalized to the mean MFI of Tax1a samples, set to 1. Plots show individual values and medians (n = 7). **E.** Jurkat cells were microporated with the pEF-Tax1-myc constructs, together with HTLV-1-LTR-Luc_Firefly_ and phRG-TK-Luc_Renilla_ reporter constructs. After 20h, Firefly and Renilla luminescence were measured in cell lysates. Plot shows Firefly luminescence normalized to Renilla luminescence, expressed as fold-change compared to the Empty vector condition set to 1 (mean with SEM, n = 3).

In order to select the Tax1 variants to be included in our study, we first collected 26 sequences of Tax1 available on Genbank at the time of the project initiation and originating from different HTLV-1 genotypes. Phylogenetic analysis of these Tax1 sequences resulted in a phylogenetic tree congruent with the phylogeny of HTLV-1 obtained in previous studies using *LTR* and *env* sequences (Fig 1A) (see [12] for a recent review). The majority of Tax1 sequences clustered in the HTLV-1a clade, reflecting the bias in enrichment of HTLV-1a sequences in public databases. As expected, the HTLV-1a-Jpn clade branched in the HTLV-1a clade. Two sequences were from the HTLV-1b Central African clade. The HTLV-1c Tax sequences were most divergent and clustered in two distinct clades: one Melanesian clade and one Australian clade. Based on this phylogenetic tree and considering reference strains previously defined as representative of the diversity of HTLV-1 [12], we selected the following Tax1 sequences for comparative analysis: the prototype Tax1a-Jpn_ATK was selected as representative of HTLV-1a sequences; Tax1b_SF26 as representative of HTLV-1b; and Tax1c-aus_CS (later referred to as Tax1c-aus) and Tax1c-mel_EM5 (later referred to as Tax1c-mel) as representative of HTLV-1c-Australia and -Melanesia, respectively. Alignment of the 4 protein sequences showed 23 sites of polymorphism spread along the 353 aa-long sequences, some of which are located in functional domains annotated in the literature on Tax1a (Fig 1B). We identified a stretch of amino acids within the C-terminal domain (residues 329–338) that is particularly variable between Tax1a and Tax1c sequences, with all polymorphic sites conserved within the Tax1c-aus and/or Tax1c-mel clades (S1A Fig). This segment is expected to adopt an extended linear conformation because it is located at the beginning of the C-terminal region of Tax (residues 326–353), which is predicted to be intrinsically disordered and elongated [33].

Selected Tax1 sequences were cloned in fusion with myc-His, myc or Flag tags into a series of plasmid and lentiviral vectors, for transient transfection or transduction, respectively, allowing ectopic expression of all variants from the same promoter and detection with the same tag. Because we initially failed to detect Tax1b protein in our first attempts (S1B Fig), we suspected a sequencing error. Indeed, analysis of Tax1b-SF26 sequence identified a single amino acid polymorphism (P_11_) uniquely found in the Tax1b-SF26 sequence, and replaced by a Leucine in other retrieved Tax1b sequences (S1B Fig, left) [34]. Directed mutagenesis of Tax1b-SF26 to introduce a Leucine at position 11 (Tax1b-SF26 P11L) in fact rescued expression (S1B Fig, right). Tax1b-SF26 P11L was therefore used in the subsequent experiments, and is later referred to as “Tax1b” for the sake of clarity.

Most experiments in our study were performed after transient microporation of tagged Tax1-encoding constructs in the Jurkat CD4^+^ T-cell line, in accordance with the natural tropism of HTLV-1 for CD4^+^ T-cells. In this system, expression of Tax1 variants constructs was first validated by western blot and flow cytometry (Figs 1C, 1D, S1C and S1D). All four Tax1a, -b, -c-mel and –c-aus variants were detected in western blot with similar band intensities. Unexpectedly, Tax1c-mel consistently migrated further in SDS-PAGE (apparent molecular weight of 38kDa) compared to Tax1a, Tax1b and Tax1c-aus (apparent molecular weight of 40kDa) (Fig 1C); this observation was independent of the cell type and of the expression vector used (S1D and S1E Fig). This might be explained by differences in the overall composition of amino acid types or in possible differences in post-translational modifications such as phosphorylation, glycosylation, acetylation, methylation, ubiquitination, and ubiquitin-like modifications, as documented previously for up to 40% of the proteome of model organisms such as yeast [35]. All four Tax1 variants were also detectable in flow cytometry, with similar percentages of Tax-positive cells (ranging from 40-60%) and mean fluorescence intensities (Figs 1D, S1D and S1E, see representative histograms on the left and data from replicates on the right). For experimental procedures requiring high percentages of positive cells, Tax-expressing Jurkat T-cells were sorted as described in the Material & Methods section and in S1F Fig, left. Post-sorting percentages of Tax-positive cells were successfully increased up to 70–90% (S1F Fig, right), confirming the efficiency of the sorting procedure.

To validate the functionality of the selected Tax1 variants transiently expressed in T-cells, we relied on an HTLV-1a LTR-driven reporter luciferase assay. Indeed, Tax-induced activation of LTR-driven transcription is critical for viral expression and well conversed among the HTLV phylogeny, as HTLV-2- and HTLV-3-encoded Tax proteins do also transactivate the viral LTR [36]. This allowed us to assume that all Tax1 variants should share the ability to activate LTR-driven transcription. Similar levels of luciferase activity were detected with all 4 variants (Fig 1E). Altogether, these results indicate that all four Tax1 variants are expressed to similar levels in cells, and are all functional for activation of LTR-driven transcription, allowing further in-depth comparison of these variants.

### Tax1 variants exhibit distinct subcellular localizations and shape distinct proximity landscapes

Previous studies have demonstrated that Tax1a sequence harbours non-conventional nuclear localization and export signals (NLS and NES, respectively), supporting its shuttling between the nucleus and the cytoplasm [37,38]. In the nucleus, Tax1a forms speckles where it interacts with host transcription and splicing factors. In the cytoplasm, Tax1a mainly associates with the surface of the Golgi apparatus, where it assembles a signalling platform scaffolded by Tax1a-bound and free poly-ubiquitin chains, known as the Tax-dependent signalosome, and required for efficient induction of NF-κB signalling [39,40]. Of note, Tax1a NLS is conserved in Tax1b and -c variants, with one differing position but involving amino acids sharing similar properties (aromatic side chains) (F20 in Tax1-a, -b and -c-mel and Y20 in Tax1c-aus, S2A Fig). In addition, key residues in Tax1a NES are also conserved (S2A Fig). These observations suggest that all variants might be able to shuttle between the nucleus and the cytoplasm.

**Fig 2:**
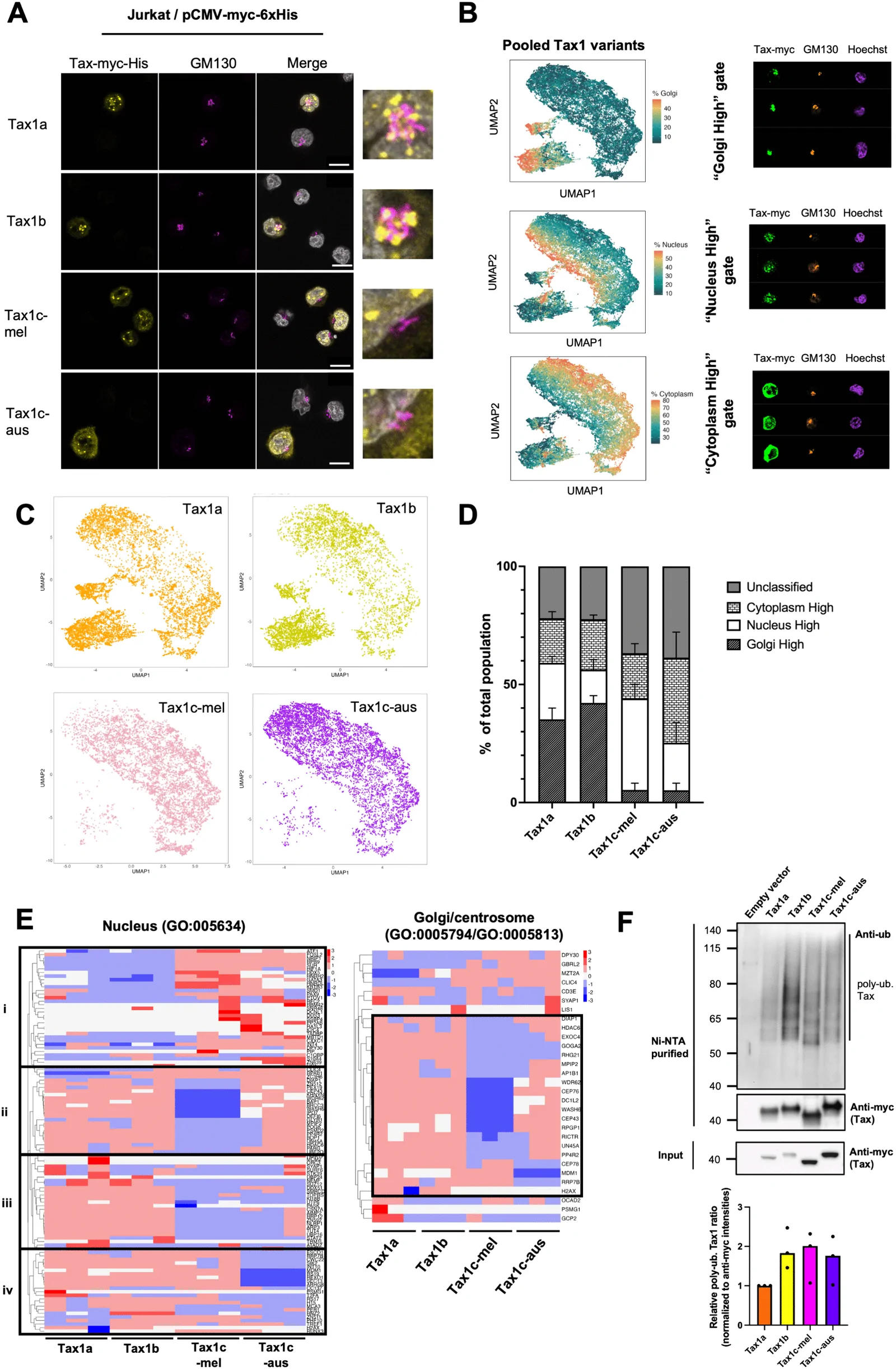
Tax1 variants exhibit distinct subcellular localizations and shape distinct proximity landscapes. **A.** Jurkat T-cells were microporated with the pCMV-Tax1-myc-6xHis constructs and cultured for 20h before analysis by immunofluorescence using anti-His (Tax, yellow) and anti-GM130 (cis-Golgi, magenta) antibodies. The right panel shows an enlarged view of the Golgi area (scale bar = 10 µm). **B.** UMAP (Uniform Manifold Approximation and Projection) analysis based on image cytometry data of Jurkat cells expressing Tax1-myc variants and stained using anti-myc (Tax, green) and anti-GM130 (cis-Golgi, orange) antibodies, together with Hoechst (purple). For each cell, the relative contribution of Tax signal in a given mask (Cytoplasm, Nucleus, Golgi) was calculated as the ratio between the total fluorescence intensity of Tax1 signal in this mask (i.e., the sum of the intensities for each pixel in the mask) on the total fluorescence intensity of Tax1 signal in the cell. UMAP was performed using fluorescence and image-based parameters (e.g., FITC intensity, area of object, relative contribution of FITC signal in each mask, Delta centroid of FITC signal, spot count of FITC signal). The UMAP plots are color-coded based on the indicated parameter value, and representative images of cells with high contribution of Tax1 signal in the Golgi (“Golgi High”), nucleus (“Nucleus High”) or cytoplasm (“Cytoplasm High”) compartment are provided. **C.** Same UMAP analysis as in (B), deconvolved based on Tax1 variant of interest. **D.** Cell frequency in subpopulations with a homogenously high contribution of Tax1 signal in the “Golgi”, the “Nucleus” or the “Cytoplasm” compartments were retrieved for independent replicates (means with SEM, n = 4). Results of statistical analysis (Friedman tests) are indicated in the text for the sake of clarity. **E.** Heatmaps indicating abundance of nuclear (left) and Golgi/centrosome (right) proteins identified as significantly enriched or depleted in the proximity of Tax1 variants compared to Tax1a, as detected by *in situ* proximity biotinylation (n = 3). Abundances were normalized by rows. Proteins with similar profiles were highlighted using the (i) to (iv) boxes. **F.** Jurkat T-cells were microporated with the pCMV-Tax1-6xHis-myc constructs and cultured for 20h before Ni-NTA purification and western blot analysis of Ni-NTA purified proteins using anti-Ub and anti-myc (Tax) antibodies (upper panels), and of total cell lysates (input) using anti-myc (Tax) antibodies (lower panel). Ub signals were quantified and normalized to myc signals in the Ni-NTA-purified fractions (n = 3 independent experiments).

To investigate the subcellular localization of Tax1 variants, we first performed immunofluorescence staining followed by confocal microscopy on microporated Jurkat T-cells (Fig 2A). As expected, all Tax1 variants were detected both in the nucleus and in the cytoplasm, with a degree of cell-to-cell heterogeneity within each condition. Tax1-positive nuclear speckles were observed with all variants, but appeared smaller and dimmer with Tax1b, and larger and brighter with Tax1c-mel, compared to Tax1a and Tax1c-aus. Strikingly, while Tax1a and Tax1b massively accumulated in the Golgi region, no substantial accumulation of either Tax1c variants in this region was observed (see magnified inset, with the GM130 Golgi marker in magenta, Fig 2A), suggesting that Tax1c might not assemble any signalosome. To quantitatively document the intra- and inter-condition heterogeneity in the subcellular localization of Tax1 variants, we developed a UMAP-based analysis of image cytometry data, that allows the unbiased clustering of cells based on multiple fluorescence and image-based features (see Material and Methods section for details). Masks for specific cellular compartments (“Golgi”, “Nucleus” and “Cytoplasm”) were determined, and the relative contributions of Tax1 signal in each of these masks were included as additional features in the UMAP analysis, as well as spot counts and Delta centroid between these masks. Fig 2B displays the UMAP of pooled data of all Tax1-positive cells from all 4 conditions (Tax1a, -b, -c-aus and -c-mel) in a representative experimental replicate. The relative contributions of Tax1 signal in each mask are color-coded in the respective panels. Based on these colour-coded UMAPs, gates corresponding to cell subpopulations with a homogenously high contribution of Tax1 signal in the “Golgi”, the “Nucleus” or the “Cytoplasm” compartments, respectively, were defined for each experimental replicate (S2B Fig), and are referred to as “Golgi High”, “Nucleus High” and “Cytoplasm High” gates, respectively (see Fig 2B for representative images of cells in these 3 gates). UMAPs from each replicate were then deconvolved based on the Tax1 variant of interest (Figs 2C and S2C), and the cell frequency in the above-defined gates was extracted (Fig 2D). Consistent with the confocal observations, the frequency of cells showing a high contribution of Tax1 signal in the “Nucleus” compartment (Fig 2D, “Nucleus High” gate) was highest for Tax1c-mel (approx. 40%), and lowest for Tax1b (approx. 15%), although these differences did not reach statistical significance (Friedman test). Again, strikingly, while 30–45% of Tax1a- and Tax1b-expressing cells showed a high contribution of Tax1 signal in the “Golgi” compartment, this was the case only for less than 3% of Tax1c-expressing cells (Fig 2D, “Golgi High” gate, p = 0.0016, Friedman test). Thus, confocal microscopy and image cytometry analysis both support a striking difference in the subcellular localization of Tax1c variants compared to Tax1a and Tax1b: (i) Tax1c-mel accumulates most frequently in the nucleus, where it assembles large Tax-nuclear speckles; and (iii) Tax1c-aus and Tax1c-mel rarely accumulate in the Golgi region.

We hypothesized that these differences in the subcellular localization could translate into distinct proximity landscapes among Tax1 variants. Proximity landscapes of Tax1 variants were obtained by *in situ* proximity biotinylation-based identification (BioID). Sequences encoding Tax1 variants were cloned in frame with the sequence of the modified bacterial biotin ligase BirA*. After confirming the expected expression and subcellular distribution of Tax1-BirA* constructs (S3A Fig), as well as their capacity to support *in situ* proximity biotinylation (S3B Fig), proteins biotinylated upon expression of Tax1 variants were purified by streptavidin pull-down, digested, and quantified by mass spectrometry. Several previously reported Tax-1a proximity partners were identified in the BirA*-Tax1a vs. BirA* comparison (e.g., SQSTM-1/p62, Itch, NFKB2-encoded p100/p52, S1 Table), validating our dataset. Proteins enriched or depleted at the proximity of a given Tax1 variant compared to Tax1a were then sorted based on the fold change (FC ≤ 0.67 or FC ≥ 1.5, *i.e., |*log_2_FC| ≥ 0.58) and significance (p-value ≤ 0.05). Among the 3109 proteins detected in the mass spectrometry analysis, 274 were identified as enriched or depleted at the proximity of Tax1b compared to Tax1a, 332 as enriched or depleted at the proximity of Tax1c-mel compared to Tax1a, and 391 as enriched or depleted at the proximity of with Tax1c-aus compared to Tax1a (S1 Table and S3C Fig). To investigate whether the differences in the subcellular localization of Tax1 variants translated into distinct proximity landscapes in the nuclear and Golgi/centrosome compartments, we then filtered the enriched or depleted proteins based on their annotation in the Gene Ontology (GO) Cell Compartments (CC) database, to select proteins annotated as localized in the nucleus, or in Golgi/centrosome compartment (Fig 2E). Interestingly, nuclear proteins among enriched or depleted proteins could be visually classified into 4 clusters: (i) proteins enriched in the proximity landscape of Tax1c (both mel and aus) compared to Tax1a and -b; (ii) proteins enriched in the proximity landscape of Tax1a, -b and -c-aus compared to Tax1c-mel; (iii) proteins enriched in the proximity landscape of Tax1a and -b compared to Tax1c (both mel and aus); and (iv) proteins enriched in the proximity landscape of Tax1a, -b and -c-mel compared to Tax1c-aus. This indicates that while Tax1a and Tax1b share most nuclear neighbours, Tax1c-aus and mel have specific nuclear proximity partners, either shared between them (i), or specific to each Tax1c variants (ii and iv), while Tax1a and Tax1b also have exclusive proximity partners not shared with Tax1c variants (iii). This suggests that Tax1 variants, while all localizing in the vicinity of nuclear partners, might assemble distinct molecular complexes. On the other hand, most of the Golgi/centrosome proteins among enriched or depleted proteins were enriched in the proximity landscape of Tax1a and b compared to Tax1c (this being particularly apparent for Tax1-mel). Since this is consistent with the image cytometry data, it validates the relevance of the BioID dataset. Altogether, this confirms that Tax1c might not assemble a Tax-dependent signalosome in association with the Golgi.

Because assembly of the Golgi-associated Tax1a signalosome depends on the poly-ubiquitinylation of Tax1 on K4 to K8 lysine residues (corresponding to lysine residues between positions 189 and 284) [41,42], we hypothesized that the inability of Tax1c to accumulate in the Golgi region might result from its inability to undergo poly-ubiquitinylation. Of note, K4 to K8 lysine residues present in Tax1a are conserved among all Tax1c variants (note that K3 -position 111- and K9 -position 324- are conserved in all Tax1 variants but Tax1c-mel). Analysis of 6xHis-tagged Tax1 variants purified on Ni-NTA beads in denaturing conditions showed stronger ubiquitinylation profiles for Tax1b variants compared to Tax1a, and ubiquitinylation profiles for Tax1c-aus and -mel variants at least as strong compared to Tax1a (Fig 2F). Thus, Tax1c inability to assemble a Golgi-associated Tax-dependent signalosome is not explained by an inefficient poly-ubiquitinylation process, but might instead be the consequence of differences in interaction with specific Golgi-associated cellular partners among the candidate proteins identified in Fig 2E.

Altogether, these observations highlight the fact that Tax1 variants exhibit specific subcellular localizations and shape specific proximity landscapes, supporting the notion that they may induce similar yet distinct signalling and transcriptional programs in T-cells.

### Tax1 variants induce a shared “core” transcriptional reprogramming in T-cells

In order to compare the transcriptional reprogramming induced by Tax1 variants, we performed bulk RNA-sequencing on Tax1-expressing Jurkat cells sorted 20 hours after microporation (see S1F Fig). An “empty control” condition (cells microporated with an empty vector) was included. To validate our dataset compared to previous data on Tax1a, we first identified differentially expressed genes (DEGs) upon Tax1a expression (compared to the “empty control” condition), based on the fold change (FC ≤ 0.67 or FC ≥ 1.5, *i.e., |*log_2_FC| ≥ 0.58) and significance (adjusted p-value ≤ 0.05). We identified 5213 DEGs upon Tax1a expression (S2 Table), among which a significant portion aligns with previously published data for both up- and down-regulated genes [30,43,44] (S4A Fig), while differences may be attributed to distinct cell types (*e.g.,* 293T, MOLT-4, primary cells) and methodologies employed (*e.g.,* microarray, bulk RNA-Seq). The up-regulated genes include the typical signature of Tax1a-expressing and HTLV-1-infected cells, such NF-κB target genes (ICAM-1, TRAF1, IL-13, IL2RA) and including NF-κB transcription factors (NFKB1, RELA, RELB, c-REL (REL)) and inhibitors (NFKBIE) (S4B Fig). Consistent with the literature, Tax1a induced DEGs in the Jun-FOS pathway (c-JUN (JUN), JUND, FOS, FOSB, FOSL1), in apoptosis resistance (BCL2L1, BCL2A1, BID), in cell cycle (EGR1, EGR2, MAD2L1) and in CREB pathway (CREBBP) (S4B Fig). Enrichment analysis of up-regulated genes highlighted the well-documented Tax1a-induced modulation of signalling pathways in T-cells, including TNF, NF-κB, JAK-STAT, and cytokine signalling pathways, as well as the dysregulation of multiple processes such as apoptosis and adhesion (S4C Fig).

**Fig 3:**
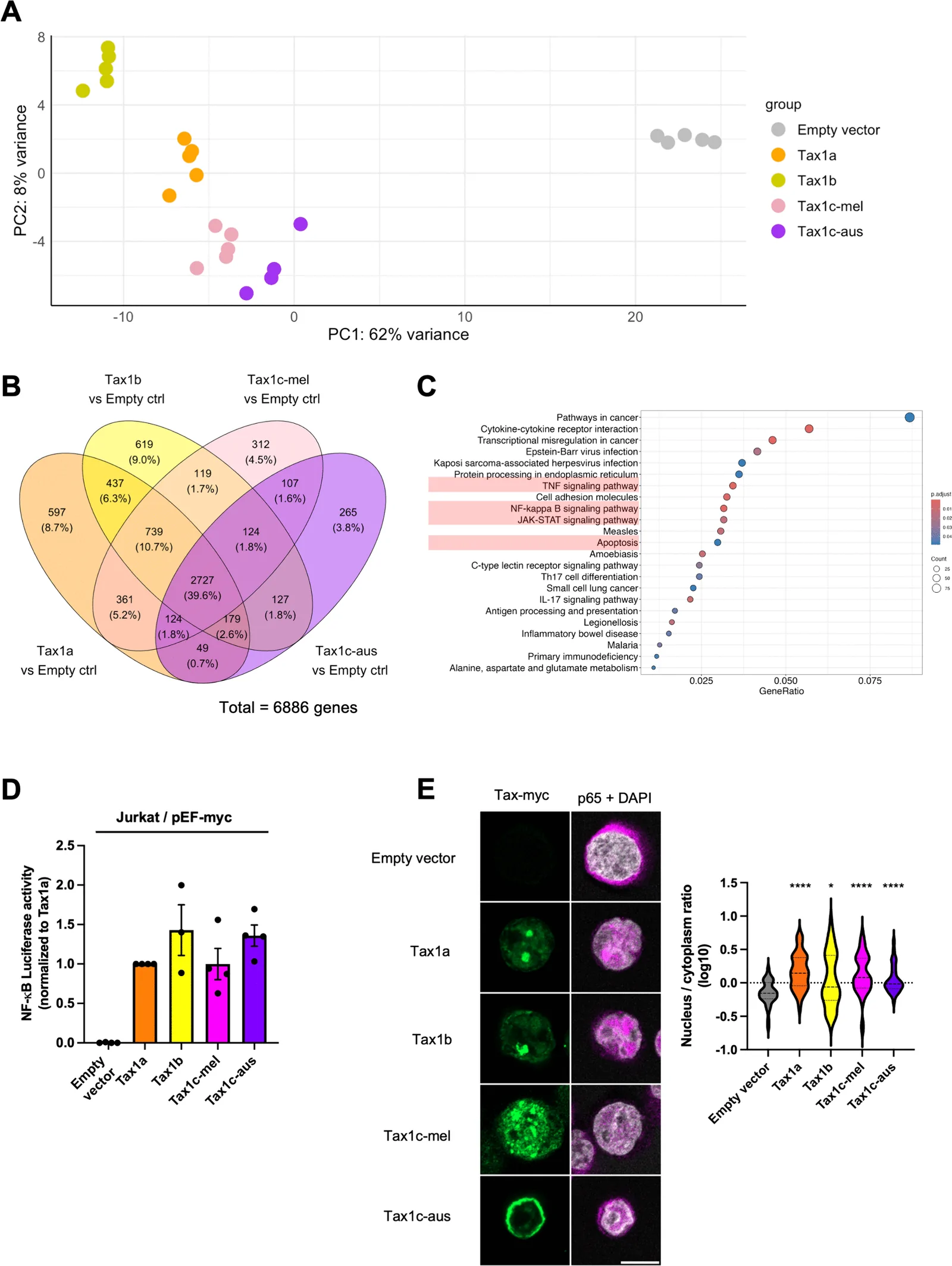
Tax1 variants induce a shared “core” transcriptional reprogramming. **A.** Principal component analysis (PCA) based on gene counts of all replicates and all Tax1 variant datasets. **B.** Venn diagram of DEGs from individual “Tax1x vs empty control” comparisons. The number of genes in each area and the corresponding percentage relative to the total DEG number (6886 genes) are indicated. **C.** Enrichment analysis of the 2727 shared DEGs between all Tax1 conditions compared to empty control. Enrichment analysis was performed across the KEGG database. **D.** Jurkat cells were microporated with pEF-Tax1-myc constructs, together with NF-κB-Luc_Firefly_ and phRG-TK-Luc_Renilla_ reporter constructs. After 20h, Firefly and Renilla luminescence were measured in cell lysates. Plot shows Firefly luminescence normalized to Renilla luminescence, expressed as fold-change compared to the Tax1a condition set to 1 (mean with SEM, n = 3-4). **E.** Jurkat cells were microporated with an empty vector or with the pCMV-Tax1-myc-6xHis constructs and cultured for 20h before analysis by immunofluorescence using anti-myc (green) and anti-p65 (magenta) antibodies (scale bar = 10 µm). The nuclei were counterstained with Hoechst (grey). For individual Tax-positive cells (or Tax-negative cells in the empty vector control), the ratio of nucleus/ cytoplasmic p65 signal was measured, and log-transformed values are depicted on the right (n = 30-50 cells per condition). Statistical analysis: Welch’s one-way ANOVA with Dunnett’s multiple comparisons test compared to Tax1a, *P < 0. 05, ****P < 0.0001.

We then compared the transcriptional programmes induced by Tax1b and Tax1c to that of Tax1a. Principal component analysis (PCA) showed that the “empty control” samples diverged most from Tax1-expressing samples, with experimental replicates of each condition clustering well (Fig 3A), confirming the good quality of our dataset for comparison analysis. We next identified the DEGs upon expression of each Tax1 variant (compared to the “empty control”, S2 Table). Of note, approximately 40% of all these DEGs were shared between variants (2727 shared DEGs among 6886 total DEGs, Fig 3B). These 2727 DEGs (which corresponded to 52% of all Tax1a-induced DEGs) were involved in cancer-related pathways, and more specifically in NF-κB activation, TNF signalling, JAK-STAT pathway, apoptosis regulation, among others, as shown by enrichment analysis across the KEGG database (Fig 3C). This suggested a shared ability of all 4 Tax1 variants to modulate these pathways. Consistently, luciferase assays as well as imaging-based p65 nuclear translocation assays revealed a similar ability of Tax1 variants to induce NF-κB activation (Fig 3D and 3E), raising the intriguing possibility that a conventional Golgi-associated signalosome is not required for efficient Tax1c-induced NF-κB activation. Altogether, this demonstrates that all Tax1 variants induce a shared “core” transcriptional reprogramming in T-cells, consistent with their sequence similarity and their shared functional domains (see Fig 1B).

### Tax1c-aus induces an additional specific transcriptional signature compared to Tax1a

**Fig 4:**
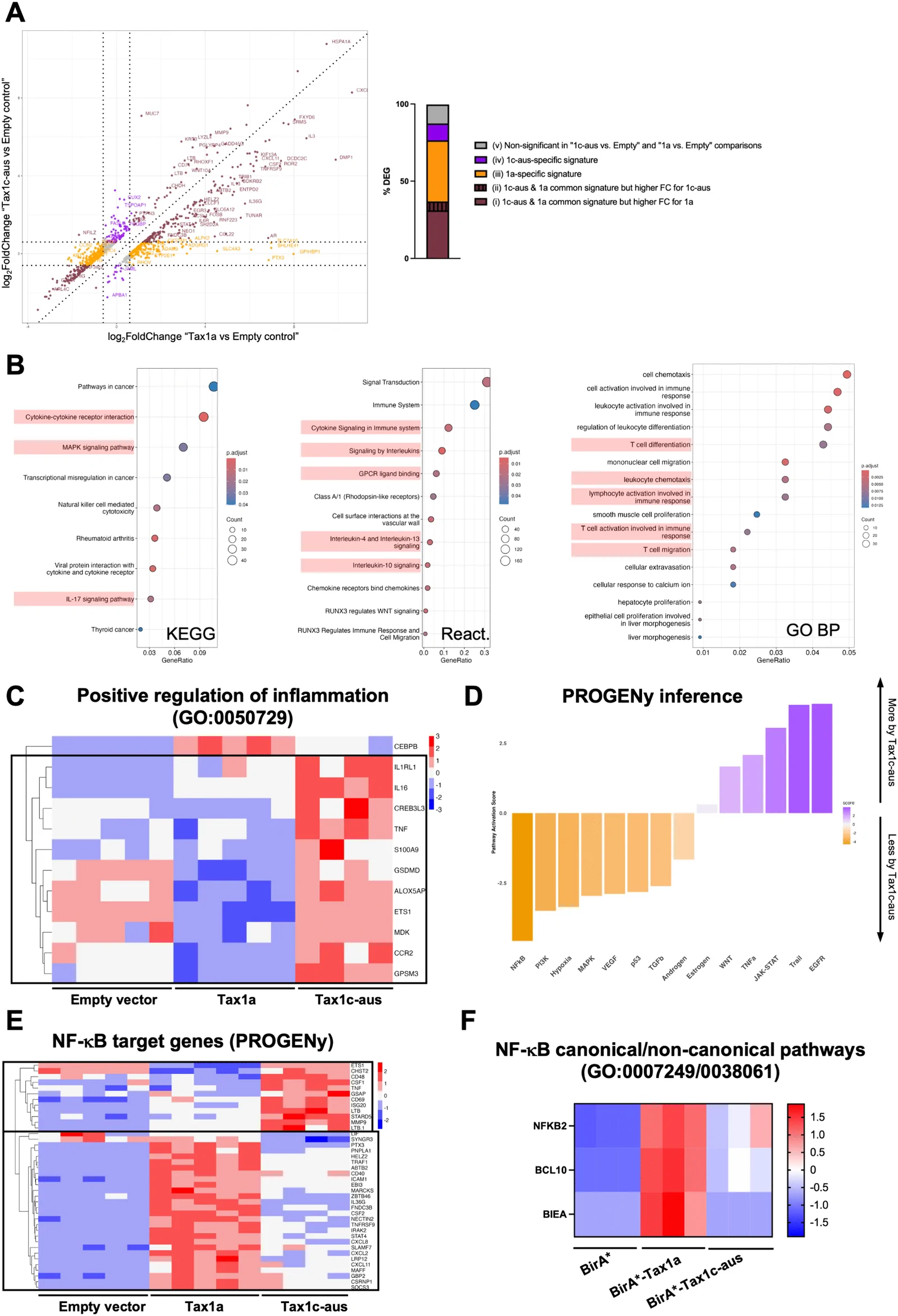
Tax1c-aus induces a specific inflammation-related transcriptional signature. **A.** Scatter plot of the 862 DEGs (|log_2_FC| ≥ 0.58 and adj. p-value ≤ 0.05) in the “Tax1c-aus vs Tax1a” comparison. The scatter plot shows the fold changes in the “Tax1a vs empty control” and “Tax1c-aus vs empty control” comparisons, in the x- and y-axis, respectively, allowing classification of the genes into 5 categories, which proportions are represented on the right (see text for additional details). **B.** Enrichment analysis of all DEGs in the “Tax1c-aus vs Tax1a” comparison. Enrichment analysis was performed across the KEGG database, Reactome pathways, and Gene Ontology Biological Processes (GO BP). **C.** Gene count heatmap of DEGs in the “Tax1c-aus vs Tax1a” comparison annotated in the “Positive regulation of inflammation” GO term. Gene counts were normalized by rows. **D.** PROGENy inference of activation score of the indicated signalling pathways. Pathways inferred as more activated in Tax1a- or Tax1c-aus-expressing samples are depicted in orange or purple, respectively. **E.** Gene count heatmap of DEGs in the “Tax1c-aus vs Tax1a” comparison annotated as genes modulated downstream of the NF-κB pathway in the PROGENy database. Gene counts were normalized by rows. **F.** Heatmap indicating the relative abundance of proteins annotated in the “NF-κB canonical pathway” and “NF-κB non-canonical pathway” GO terms and enriched or depleted in the proximity of Tax1c-aus compared to Tax1a, as detected by *in situ* proximity biotinylation (n = 3). Abundances were normalized by rows.

Because the shared DEGs represent only approx. 40% of all DEGs in our dataset, we further investigated the nature of the specific transcriptional signatures induced by Tax1 variants, compared to Tax1a, starting with Tax1c-aus. We identified 862 DEGs upon Tax1c-aus expression, compared to Tax1a expression (|log_2_FC| ≥ 0.58 and adj. p-value ≤ 0.05, S2 Table), that were further plotted on a scatter plot (x-axis: log_2_FC in the Tax1a vs “empty control” comparison; y-axis: log2FC in the Tax1c-aus vs “empty control” comparison) to classify them into 5 categories (Fig 4A): (i) deregulated both by Tax1c-aus and Tax1a compared to the “empty vector” condition, but significantly more by Tax1a (31.1% of the 862 “Tax1-aus vs Tax1a” DEGs, brown); (ii) deregulated both by Tax1c-aus and Tax1a compared to the “empty vector” condition, but significantly more by Tax1c-aus (5.8% of DEGs, hatched brown); (iii) deregulated by Tax1a compared to the “empty vector” condition, but not by Tax1c-aus (39.6% of DEGs, orange); (iv) deregulated by Tax1c-aus compared to the “empty vector” condition, but not by Tax1a (10.9% of DEGs, purple); or (v) not significant in the Tax1c-aus vs. “empty vector” and Tax1a vs. “empty vector” comparisons (grey). This reveals that, in addition to the shared “core” genes modulated by both Tax1a and Tax1c-aus – although quantitatively different, Tax1c-aus also induces a specific transcriptional signature, characterized by the up- or down-regulation of a Tax1c-aus-specific gene set, and by the absence of modulation of a gene set otherwise modulated by Tax1a.

To comprehensively investigate the processes in which these 862 “Tax1c-aus vs Tax1a” DEGs (both up- and down-regulated) are involved, enrichment analysis was performed using the KEGG database, as well as the Reactome pathways and GO Biological Processes (BP) databases (Fig 4B). Signalling pathways including cytokine signalling (and especially interleukin signalling such as IL-10, IL-13, IL-4 and IL-17), GPCR signalling, and MAPK signalling, were among significantly enriched terms, consistent with enriched immune processes including inflammatory response, T-cell activation, chemotaxis, and T-cell migration (Fig 4B). Of note, among the 12 DEGs annotated in the “positive regulation of inflammatory response” term (Gene Ontology GO:0050729), 11/12 were significantly up-regulated by Tax1c-aus compared to Tax1a (IL1RL1, IL16, CREB3L3, TNF, S100A9, GSDMD, ALOX5AP, ETS1, MDK, CCR2, GPSM3, Fig 4C), suggesting a higher pro-inflammatory capacity of Tax1c compared to Tax1a.

We next asked which signalling pathways could best account for this “Tax1c-aus vs Tax1a” differential transcriptional signature. We used the PROGENy database [45] to infer the activation of cellular signalling pathways based on the transcript count tables from Tax1a- and Tax1c-aus-expressing samples. Interestingly, this analysis inferred a trend for a lower level of activation of the NF-κB signalling pathway in the Tax1c-aus- vs Tax1a-expressing samples (Fig 4D), in apparent contradiction with the luciferase assays afore mentioned (see Fig 3D). A closer investigation of the list of “Tax1c-aus vs Tax1a” DEGs allowed us to retrieve 40 genes modulated downstream of NF-κB pathway, among which the majority (28/40) were less activated by Tax1-aus compared to Tax1a (Fig 4E). Of note, a subset of NF-κB-modulated genes (12/40) was in fact found to be more activated by Tax1c-aus compared to Tax1a, including genes encoding proinflammatory cytokines (CSF1, TNF, LTB) and T-cell activation markers (ISG20, CD48 and CD69).

Filtering the proximity labelling data for proteins enriched in the vicinity of Tax1a compared to Tax1c-aus and involved in NF-κB signalling identified p100 (*NFKB2*), BCL10 and BIEA (Fig 4F), hinting at a change in the quality of NF-κB signalling. Such a change in the quality of NF-κB signalling, rather than a binary on/off activation status, might explain why no significant differences could be observed using proxies such as luciferase assays and translocation assays (see Fig 3D and 3E).

Altogether, these results show that in addition to a “core” transcriptional reprogramming shared with Tax1a, Tax1c-aus also induces a specific pro-inflammatory transcriptional signature, driven in part by a differential activation of the NF-κB signalling pathway. We postulate that differential NF-κB signalling might be a direct consequence of the limited localization of Tax1c at the Golgi.

### Tax1c-mel also induces a specific transcriptional signature compared to Tax1a, driven in part by a differential activation of the NF-κB signalling pathway

We next asked whether Tax1c-mel also induced a specific transcriptional signature compared to Tax1a. Following the same analysis pipeline described above for Tax1c-aus, we identified 261 DEGs upon Tax1c-mel expression, compared to Tax1a expression (|log2FC| ≥ 0.58 and adj. p-value ≤ 0.05, S2 Table), that could further be classified into the same 5 categories (S5A Fig): (i) deregulated both by Tax1c-mel and Tax1a compared to the “empty vector” condition, but significantly more by Tax1a (31.8% of the 261 “Tax1-mel vs Tax1a” DEGs); (ii) deregulated both by Tax1c-mel and Tax1a compared to the “empty vector” condition, but significantly more by Tax1c-mel (18.4% of DEGs); (iii) deregulated by Tax1a compared to the “empty vector” condition, but not by Tax1c-mel (25.7% of DEGs); (iv) deregulated by Tax1c-mel compared to the “empty vector” condition, but not by Tax1a (14.6% of DEGs); or (v) not significant in the Tax1c-mel vs. “empty vector” and Tax1a vs. “empty vector” comparisons (grey).

Enrichment analysis performed on all these 261 DEGs highlighted pathways already described with Tax1c-aus, such as interleukin signalling (including IL-10, IL-4, IL-13) and GPCR signalling pathways (S5B Fig), supporting the notion that both Tax1c-aus and -mel induce similar transcriptional signatures at the functional level. Similar to Tax1c-aus, PROGENy analysis inferred a lower level of activation of the NF-κB signalling pathway in the Tax1c-mel- vs Tax1a-expressing samples (S5C Fig). Again, among the 22 NF-κB-modulated genes identified among the “Tax1c-mel vs Tax1a” DEGs, 16/22 were less activated by Tax1-mel compared to Tax1a (most of which were also found in the Tax1c-aus analysis, see Fig 4E), and 6/22 were more activated by Tax1c-mel compared to Tax1a, including the gene encoding the proinflammatory cytokine (CSF1) (S5D Fig).

### Tax1b modulates the expression of a similar gene set compared to Tax1a, but with a higher magnitude

**Fig 5:**
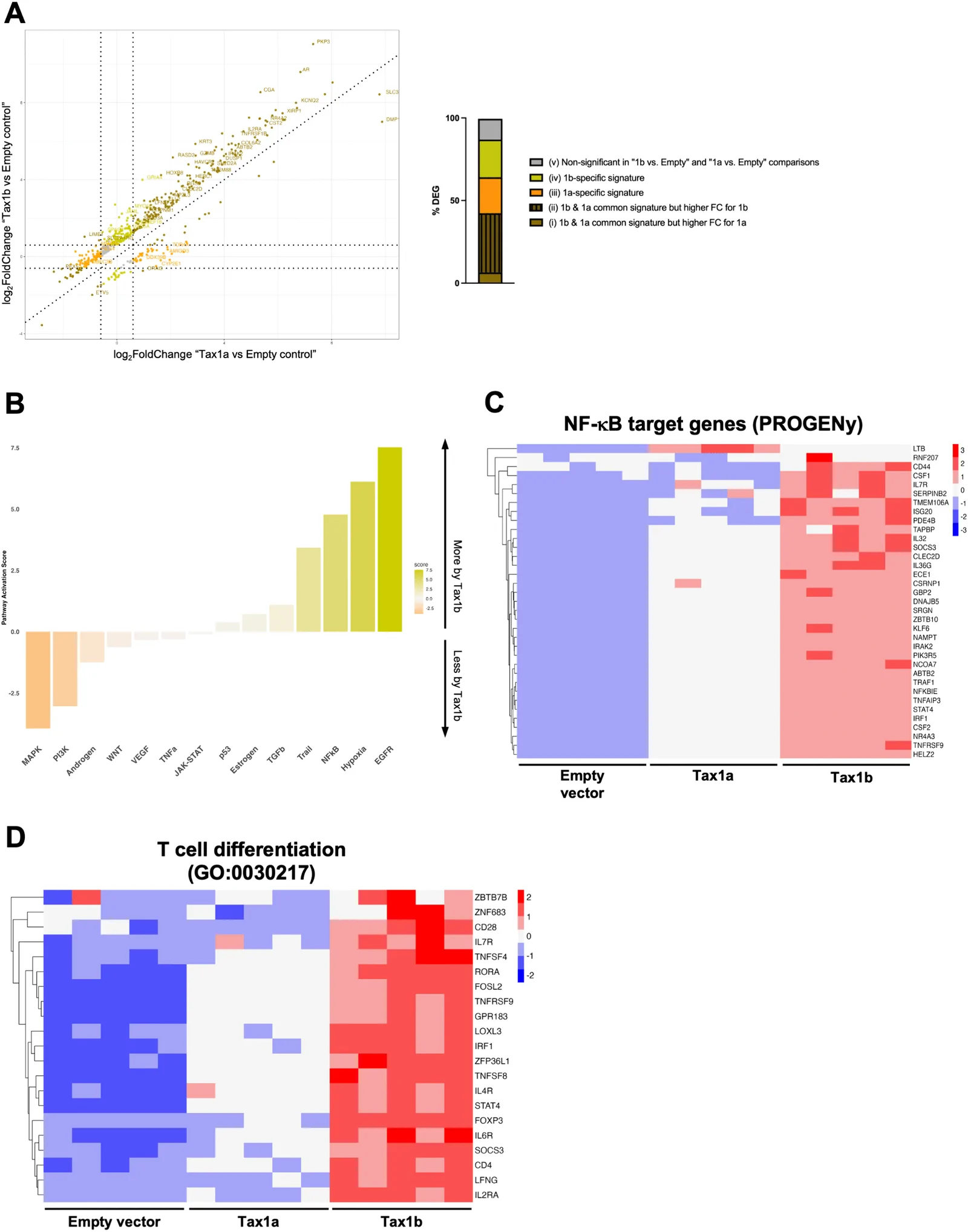
Tax1b modulates the expression of a similar gene set compared to Tax1a, but with a higher magnitude. **A.** Scatter plot of the 509 DEGs in the “Tax1b vs Tax1a” comparison. The scatter plot shows the fold changes in the “Tax1a vs empty control” and “Tax1b vs empty control” comparisons, in the x- and y-axis, respectively, allowing classification of the genes into 5 categories, which proportions are represented on the right (see text for additional details). **B.** PROGENy inference of activation score of the indicated signalling pathways. Pathways inferred as more activated in Tax1a- or Tax1b-expressing samples are depicted in orange or green, respectively. **C.** Gene count heatmap of DEGs in the “Tax1b vs Tax1a” comparison annotated as genes modulated downstream of the NF-κB pathway in the PROGENy database. Gene counts were normalized by rows. **D.** Gene count heatmap of DEGs in the “Tax1b vs Tax1a” comparison annotated in the “T-cell differentiation” GO term. Gene counts were normalized by rows.

We then performed a similar comparative transcriptomics analysis between Tax1b- and Tax1a-expressing T-cells and identified 509 DEGs (S2 Table). In stark contrast with Tax1c-aus and -mel analysis (see Figs 4A and S5A), most of these 509 DEGs were either deregulated both by Tax1b and Tax1a compared to the “empty vector” condition, but significantly more by Tax1b (35.8% of the 509 “Tax1b vs Tax1a” DEGs, (ii) category), or, to a lesser extent, deregulated by Tax1b compared to the “empty vector” condition, but not by Tax1a (22.8% of DEGs, (iv) category, Fig 5A). Only 21.8% of DEGs were deregulated by Tax1a compared to the “empty vector” condition, but not by Tax1b (Fig 5A, (iii) category), and these genes showed only modest fold changes over the “empty vector” condition upon Tax1a expression. This indicates that Tax1b modulates the expression of a similar gene set compared to Tax1a, but with a higher magnitude. Given that both variants showed a comparable expression at the protein level (see Fig 1C and 1D), this suggests that Tax1b is driving transcriptional changes more efficiently than Tax1a.

Using PROGENy on the “Tax1b vs Tax1a” comparison, we inferred that NF-κB, Hypoxia and EGFR pathways were more activated by Tax1b compared to Tax1a, while MAPK and PI3K pathways were less activated (Fig 5B). Consistent with these inferences, analysis of the expression level of NF-κB target genes showed that all but 1 NF-κB target genes among “Tax1b vs Tax1a” DEGs were over-expressed by Tax1b compared to Tax1a (Fig 5C), supporting the notion that the strong association of Tax1b with the Golgi apparatus described in Fig 2 could allow a more efficient activation of the NF-κB pathway. Also, filtering the proximity labelling data for proteins involved in NF-κB signalling identified NF-κB modulators (IRAK1, ILK, PHB2, TERF2IP) among proteins enriched in the vicinity of Tax1b compared to Tax1a, as well as NF-κB inhibitors (RBCK1) among proteins depleted in the vicinity of Tax1b compared to Tax1a (S1 Table).

When analysing the DEG list, we noted that the ATL-specific *FOXP3* and *IL2RA* genes were among genes over-expressed by Tax1b compared to Tax1a. We thus specifically analysed the expression of genes involved in the T-cell differentiation process (GO:0030098). We observed that all genes involved in T-cell differentiation among “Tax1b vs Tax1a” DEGs were over-expressed by Tax1b compared to Tax1a (Fig 5D)*.* Altogether, these observations support the notion that Tax1b-induced signature, although closely resembling the one induced by Tax1a, consists in a stronger modulation of target genes, possibly through the marked accumulation of Tax1b at the Golgi apparatus, thereby strengthening the polarization of T-cells towards a Treg-like phenotype as observed in ATL cells [46].

### Tax1 variants activate distinct signalling cascades, as revealed by kinomics analysis

**Fig 6:**
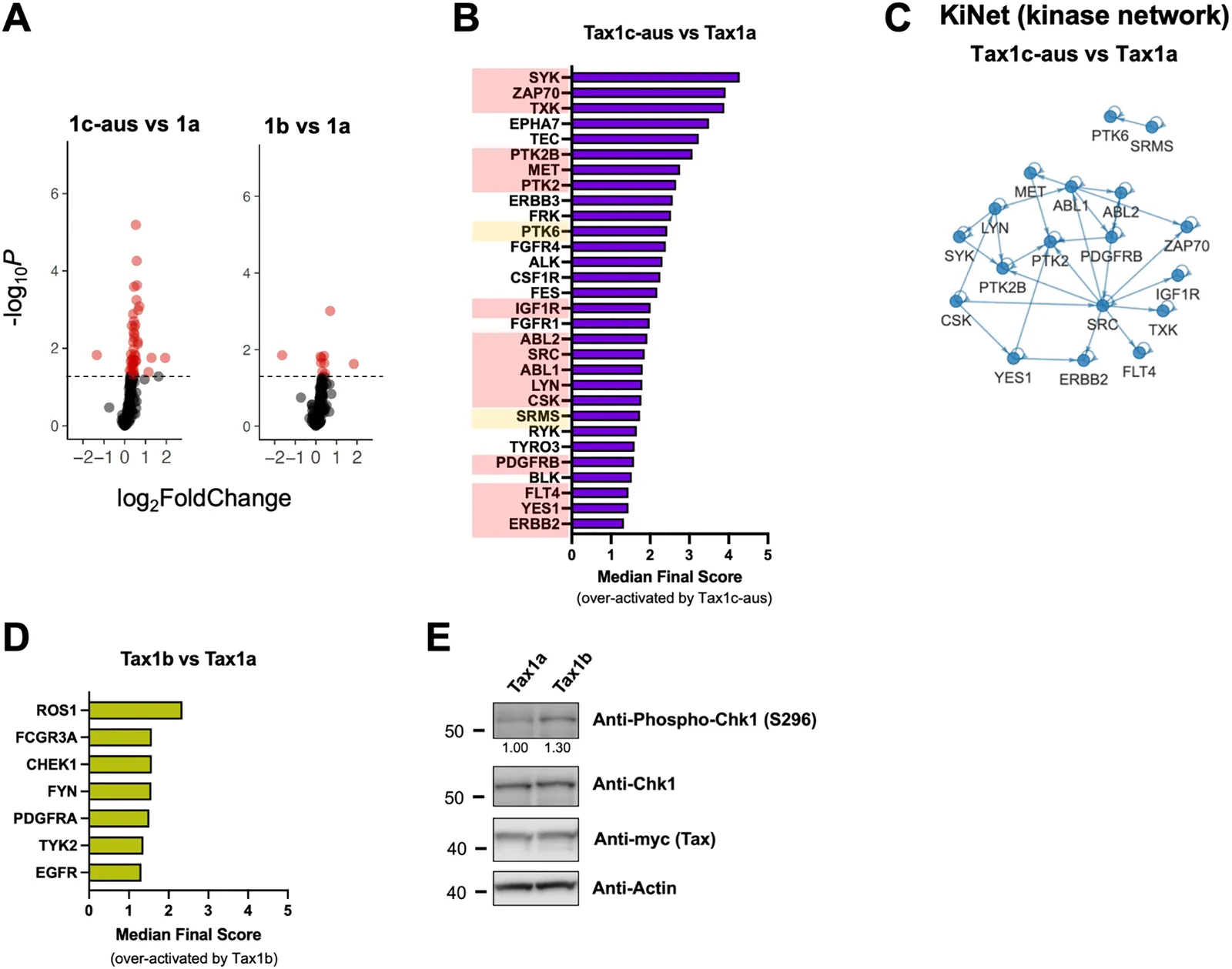
Tax1c-aus and Tax1b induce specific kinomics signatures. **A**. Volcano plots of the differentially phosphorylated PTK phosphosites in the “Tax1c-aus vs Tax1a” and “Tax1b vs Tax1a” comparisons. In red are phosphosites that show significant differences compared to the Tax1a reference condition (p-value ≤ 0.05). **B.** Upstream kinase analysis of the “Tax1c-aus vs Tax1a” comparison restricted to tyrosine kinases differentially activated by Tax1c-aus compared to Tax1a, but not by Tax1b. Kinases represented in **(C)** are highlighted in red and orange. **C.** Network analysis of the kinases shown in **(B)** using the KiNet visualization interface. Kinases with no connection to other kinases of the list are not displayed. Arrows indicate the “kinase-to-substrate” relationships among included kinases, highlighting regulatory networks. **D.** Upstream kinase analysis of the “Tax1b vs Tax1a” comparison restricted to tyrosine kinases differentially activated by Tax1b compared to Tax1a, but not by Tax1c-aus. **E.** Tax1a- and Tax1b-expressing cells were analyzed by western blot using anti-phospho-Chk1 (S296) and anti-Chk1 antibodies.

In order to pinpoint differential activation of signalling cascades by Tax1 variants that could be related to the specific transcriptional signatures described above, we performed comparative multiplex kinome activity profiling in Jurkat T-cells. The assay included a panel of 144 serine/ threonine kinase (STK) and 196 tyrosine kinase (PTK) substrates. After incubation of cell lysates on the phosphosite microarrays, phosphorylated sites were detected by fluorescently labelled antibodies. Statistical comparison of phosphorylated phosphosites abundance in each sample identified 44 differentially phosphorylated PTK phosphosites in the “Tax1c-aus vs Tax1a” comparison, and 11 in the “Tax1b vs Tax1a” comparison (Fig 6A). In contrast, the number of differentially phosphorylated STK phosphosites was found to be below standard significance for both comparisons (<8 significant phosphosites per comparison). Based on these differentially phosphorylated phosphosites, upstream kinase analysis inferred 67 and 44 tyrosine kinases differentially activated by Tax1c-aus and Tax1b, compared to Tax1a, respectively (|median final score| > 1.3, S3 Table).

On the one hand, we specifically focused on the tyrosine kinases that were inferred as differentially activated by Tax1c-aus compared to Tax1a, but that were not differentially activated by Tax1b compared to Tax1a (Fig 6B). All tyrosine kinases inferred as differentially activated specifically by Tax1c-aus were over-activated in Tax1c-aus-expressing cells compared to Tax1a-expressing cells (median final score >0). Interestingly, network analysis using the KiNet visualization interface [47] revealed that several of these kinases, including Lyn, Syk and ZAP70, were linked to activation of the TCR (Fig 6C), that could be consistent with the transcriptional signature described above showing an enrichment in terms related to T-cell activation (see Fig 4B and 4E).

On the other hand, filtering tyrosine kinases that were inferred as differentially activated by Tax1b compared to Tax1a, but that were not differentially activated by Tax1c-aus compared to Tax1a, highlighted a very limited number of kinases, all over-activated by Tax1b, with no apparent enrichment in a given pathway (Fig 6D). We noted the presence of CHEK1, which encodes Chk1. Chk1 is known to auto-phosphorylate on residue S296 upon activation. We therefore compared the levels of Chk1 S296 phosphorylation and observed a 1.3-fold increase in Tax1b-expressing cells compared to Tax1a-expressing cells (Fig 6E), in agreement with the kinomic analysis.

Chk1 is a kinase involved in checkpoint-mediated cell cycle arrest and activation of DNA repair. These data are consistent with the proximity landscape of Tax1b obtained by proximity biotinylation, with p53-binding proteins including DAXX and TRIM24, as well as chromosome maintenance proteins (CHD1, MCM2, H2AX), being enriched in proximity of Tax1b compared to Tax1a (S1 Table). Taken together, these data suggest that compared to Tax1a, Tax1b could exert a specific control over DNA damage and cell cycle-related pathways.

### Tax1 variants show distinct efficiency in modulating the cell cycle and inducing cell transformation

**Fig 7:**
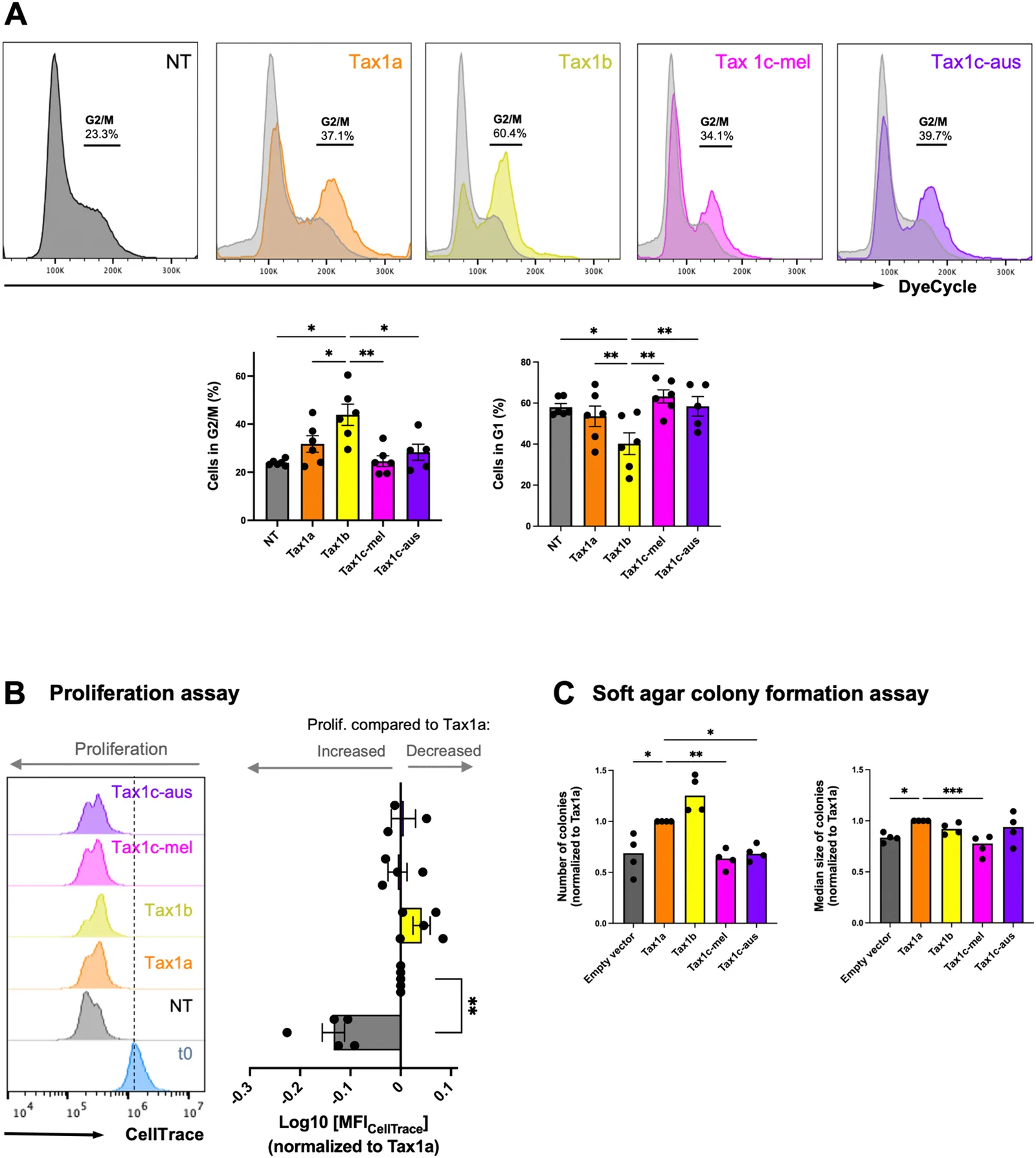
Tax1 variants exhibit distinct cell cycle modulation and cell transforming capacity. **A.** Jurkat T-cells were transduced with Tax1-myc-expressing lentiviruses or left without transduction (NT: “No transduction”), and cultured for 48h before analysis by flow cytometry using the DNA intercalant DyeCycle and anti-myc antibodies. The histograms show a representative replicate (top panel). For Tax1 conditions, overlays are shown, with Tax1-positive cells in their respective colours and Tax-negative cells of the same sample in light grey. The gating strategy is shown in S6B Fig. Gates and percentages of cells in G2/M are shown on the histograms (only for Tax1-positive cells for the Tax1 conditions). Percentages of cells in G2/M and in G1were quantified for 5 independent replicates (bottom panels, mean with SEM). Statistical analysis: mixed-effect analysis with Tukey’s multiple comparisons test, *P < 0. 05, **P < 0.01. **B.** Jurkat T-cells were stained with Deep Red CellTrace, transduced after 24h and cultured for 48h before analysis by flow cytometry using anti-myc antibodies. The histogram shows a representative replicate, with t0 corresponding to cells fixed 24h after CellTrace staining (left panel). CellTrace MFI was quantified for 3-5 independent replicates and normalized to Tax1a (right panel, mean with SEM). Statistical analysis: mixed-effect analysis with Dunnett’s multiple comparisons test, **P < 0. 01. **C.** NIH Rat-1 cells were transfected with pCMV-Tax1-myc-6xHis constructs and cultured for 20h. Cells were then transferred into soft-agar for 6-8 days before microscopy analysis. Wells were scanned and each colony was pictured. The number of colonies (normalized to Tax1a) was plotted for 4 independent replicates. The median size of colonies was plotted for 3 independent replicates. Plots show individual and median values. Statistical analysis: repeated-measures one-way ANOVA with Dunnett’s multiple comparisons test compared to Tax1a, *P < 0. 05, **P < 0.01.

Because the kinomics and proximity labelling data suggested that compared to Tax1a, Tax1b could exert a specific control over DNA damage and cell cycle-related pathways, we set up a series of functional assays to compare the cell cycle modulation by Tax1 variants. To do so, we produced lentiviral vectors encoding the different Tax1 variants and used these lentiviral vectors to transduce Jurkat T-cells. All four Tax1 variants were detectable in flow cytometry, with similar percentages of Tax-positive cells and mean fluorescence intensities (S6A Fig). Using this system, we first assessed by flow cytometry the distribution of cells in the cell cycle, 48h post-transduction (see S6B Fig for the gating strategy). Interestingly, Tax1b-expressing cells accumulated in the G2/M phase of the cycle, with a two-fold increase in the proportion of cells in the G2/M phase and a 1.5-fold decrease in the proportion of cells in the G1 phase, when compared to non-transduced cells (Fig 7A). Although not significant, Tax1a also seemed to induce a slight accumulation of cells in G2/M compared to non-transduced cells. To assess whether this observed accumulation in G2/M was due to a blockade in the cell cycle, we performed a proliferation assay (Fig 7B). Compared to non-transduced cells, all Tax-expressing cells showed a reduced proliferation rate, thus confirming a blockade in the cell cycle. When compared to Tax1a, we noted that the proliferation of Tax1b-expressing cells tended to be reduced, although this did not reach statistical significance (Fig 7B). Next, we wondered whether the differential arrest in G2/M between variants as well as the differential activation of Chk1 (see Fig 6D and 6E) could be a consequence of differential induction of DNA damage. Thus, 48h post-transduction of Tax1, we stained cells for γH2AX, a marker for double-strand breaks (DSB). All Tax1 variants induced an increased γH2AX MFI both in the G1 and G2 phases when compared to non-transduced cells, with no significant differences among Tax1 variants (S6C Fig). Altogether, we showed that Tax1b tends to induce a more efficient cell-cycle arrest than Tax1a and Tax1c, in the absence of significant differential induction of DSB. Together with the over-activation of Chk1 inferred from kinomics analysis, this suggests that DSB checkpoint activation might be more efficient in Tax1b-expressing cells, leading to a more efficient cell-cycle arrest.

Early G2/M arrest was previously used as a proxy for oncogenic potential for Tax1a, which prompted us to assess the transforming capacity of Tax1 variants with a standard NIH Rat-1 soft agar colony formation assay previously used for Tax1a and Tax2 [17,18] (see S1E Fig for comparison of Tax1 expression levels in Rat-1 cells). We quantified the number and size of colonies after 6–8 days (Fig 7C). Interestingly, Tax1b induced 1.25 times more colonies than Tax1a, while Tax1c-aus and -mel induced 1.5 to 2 times less colonies than Tax1a, the differences being significant (Fig 7C). Moreover, we observed a consistent 1.3- to1.6-fold reduction of colony median size in Tax1c-mel conditions compared to Tax1a, while the size of Tax1c-aus-induced colonies was similar to Tax1a-induced colonies (Fig 7C). Taken together, these results support the notion that in this experimental setting, Tax1b has a slightly higher transforming capacity than Tax1a, while both Tax1c-aus and -mel have a lower transforming capacity.

## Discussion

In this work, we hypothesized that HTLV-1 genotypes may present different pathogenic properties [7]. Focusing on the major viral determinant Tax1, and using image cytometry combined to proximity labelling, transcriptomics and kinomics, we provide the first comprehensive comparative analysis of Tax1 variants from genotypes a (Japanese), b (African) and c (Australo-Melanesia) expressed in Jurkat T-cells. As expected by the level of amino acid identity, our results indicate that Tax1b closely parallels Tax1a functional features at the cellular level, with a higher magnitude, while Tax1c harbours divergent features. Indeed, Tax1c induces a specific transcriptional signature that could be driven in part by a specific quality of NF-κB signalling, correlated to a limited localisation of Tax1c to the Golgi/centrosome. The transcriptional program induced by Tax1c appears as biased towards activation of T-cells and positive regulation of inflammation, consistent with the specific activation of kinases involved in TCR signalling, and contrasting with the bias towards an ATL/Treg-like signature observed upon Tax1b expression [46]. Importantly, the transforming capacity of Tax1c was decreased compared to Tax1a, while that of Tax1b was increased, overall suggesting that while Tax1a and Tax1b could favour oncogenic signalling, Tax1c could rather favour inflammatory signalling. Although translation into pathophysiological mechanisms upon infection by either of HTLV-1 genotypes should be made with great caution, these observations are consistent with the pre-clinical and clinical reports indicating an over-representation of inflammatory manifestations (in particular bronchiectasis), and an under-representation of malignant manifestations, in people living with HTLV-1c compared to HTLV-1a [7,48], as well as in animal models of the infection [10,11]. Of note, specific clinical data on the HTLV-1c-mel subtype are very scarce to date. Epidemiological studies in Melanesia were conducted mostly on the general healthy population in the Vanuatu Islands [49] and New Caledonia [50]. Only two cases of HAM/TSP were reported in the Salomon Island [49].

Because HTLV-1 has long been considered as genetically stable compared to other retroviruses, functional comparisons among viral proteins derived from distinct HTLV-1 genotypes have attracted little attention until recently. Nonetheless, the notion that closely related Tax1 variants could harbour specific functional features was supported by two previous studies comparing Tax1a from the Japanese and the transcontinental HTLV-1a subgroups, that reported specific functional reprogramming induced in T-cells by these two variants despite their very close sequence similarity [51,52]. Thus, specific functional features of more distantly related Tax1 variants such as Tax1b and Tax1c were to be expected, consistent with the observations that we report here.

The first striking result of our work is that compared to Tax1a and Tax1b, Tax1c variants are not associated to the Golgi apparatus, as demonstrated by confocal microscopy and image cytometry, and as confirmed by proximity labelling. This raises questions as to (i) what are the causes of such a distinct subcellular localization; and (ii) what are the consequences. Tax1a sequence contains 10 lysine residues, referred to as K1 to K10. Tax1a association with the Golgi apparatus has been thoroughly demonstrated to rely on Tax1a poly-ubiquitinylation on K4 to K8 lysine residues (lysine residues between positions 189 and 284) [53,54], which allows Tax to interact with Golgi-associated CADM1 [55], OPTN, TAX1BP1 and SQSTM1/p62 [56,57], among other partners, leading to the assembly of an NF-κB-activating signalosome. However, Tax1c did not show any detectable defect in their poly-ubiquitinylation status, suggesting that for Tax1c poly-ubiquitinylation is not sufficient to drive the accumulation at the Golgi. Further investigation is however required to formally demonstrate that Tax1c variants undergo the same topology of poly-ubiquitinylation compared to Tax1a, which was not addressed here. Consistently, proximity labelling identified a set of Golgi-associated proteins that were depleted in the vicinity of Tax1c, compared to Tax1a. The depletion of these proteins in the proximity network of Tax1c could simply reflect the poor accumulation of Tax1c in this compartment, without any causal relationship. Alternatively, the inability of Tax1c to interact with one or several of these proteins could explain the difference in Tax1c vs. Tax1a sub-cellular localisation. Should this be the case, more knowledge is needed to understand the process of Tax1a trafficking from the site of translation to the peri-Golgi area. Of note, in our transcriptomics data set, we observed that some genes involved in Golgi/ER transport are down-regulated by Tax1c compared to Tax1a (SEC24A, SEC24D, TMED7, COPA, GOLGB1, ANK2, RAB1A). We could thus speculate that these proteins may be involved in the trafficking of Tax1 to the peri-Golgi area, a mechanism that could be hindered upon transcriptional down-regulation in Tax1c-expressing cells. It is also noteworthy that Tax1c accumulate polymorphisms in the C-terminal domain (see S1A Fig), including in the DHE motif previously shown to contribute to Tax1a interaction with the secretory pathway [58]. Whether these polymorphisms could also take part in the distinct localization pattern would warrant further investigation.

What could be the functional consequences of the lack of accumulation of Tax1c at the surface of the Golgi apparatus? Tax1a is known to constitutively activate the NF-κB signalling pathway by recruiting intermediates such as the modulator IKKγ/NEMO to the Golgi signalosome [25,53,54,59]. Despite the absence of such a signalosome, we showed that Tax1c variants still activate the NF-κB pathway, hinting at an alternative activation mechanism. Similar observations have been made with the Tax protein from HTLV type 2 (Tax2), which does not accumulate at the Golgi but still constitutively activates the canonical NF-κB pathway [40,60–62]. It is striking to observe that Tax1c-aus has a similar localization to that of Tax2, with most of the signal located in the cytoplasm. There are however differences between these proteins, since Tax2 was shown to be poorly poly-ubiquitinylated (despite Tax1a lysines K4 to K8 being conserved in the Tax2 sequence) [62], which is in contrast to Tax1c which undergoes efficient poly-ubiquitinylation. Taken together, these observations indicate a degree of flexibility in the mechanisms of NF-κB activation by Tax proteins from different HTLV genotypes or types, with varying degrees of dependency on Tax poly-ubiquitinylation or Golgi accumulation, and call for caution when generalizing observations from one variant to the other. In addition, such a flexibility in the mechanisms of Tax-dependent NF-κB activation could relate to specific qualities of NF-κB signalling induced by these variants, consistent with the observation that Tax1 and Tax2 do not activate the same NF-κB dimers or target genes for instance [43,63,64]. Of note, the NF-κB subunit p100 was among the proteins depleted in proximity of Tax1c compared to Tax1a in our proximity labelling data, and whether Tax1c differently activate the non-canonical NF-κB pathway compared to Tax1a and Tax2 is currently being tested. Pathway inference from our transcriptomics data on Tax1a- and Tax1c-expressing cells indeed supported the notion that differences in the quality of NF-κB signalling may account, at least in part, for the distinct transcriptional signature of the proteins by specifying distinct subsets of NF-κB target genes either up- or down-regulated by Tax1c compared to Tax1a and to the control condition. In addition to the broad canonical vs non-canonical NF-κB dichotomy, several mechanisms have been shown to specify the subsets of NF-κB target genes that are responsive in a given context [65]. Some of these mechanisms rely on chromatin modifications that are removed on specific NF-κB responsive genes under specific stimuli to enhance transcription. In our proximity labelling dataset, we identified demethylases and acetylases enriched or depleted in the vicinity of specific Tax variants. These include PHF2, a H4K20me3 demethylase known to remove repressive marks on specific NF-κB target genes [66], which we observed exclusively in the proximity landscape of Tax1c-aus. NKIRAS2, which has been described as contributing to the specificity of NF-κB signature [67,68], could also be involved. When articulated together with the enrichment analyses and with the behaviour of Tax1a and Tax1c in transformation assay, these data support the notion that while each variant activates NF-κB signalling, Tax1a might favour oncogenic signalling while Tax1c might rather favour inflammatory signalling, possibly through specific cooperation with other transcription factors such as AP-1 downstream of MAPK pathway. This offers a unique opportunity to gain a better understanding on how subtle changes in interaction with host signalling pathways could influence the output transcriptional fate of the cell, a knowledge that could be leveraged beyond the field of HTLV.

As stated above, Tax1b closely parallels Tax1a functional features at the cellular level but seems to do so with a higher magnitude. Tax1b strongly accumulates at the Golgi, and increases expression of NF-κB target genes when compared to Tax1a, even though this could not be detected in the luciferase reporter gene assay. This higher efficiency of Tax1b was associated with a bias towards an ATL-like signature, with up-regulation of FOXP3 [69,70], IL2RA [71], CD4, IL4R [72] and STAT4 [73] compared to Tax1a. Interestingly, Tax1a has been showed to modify the cell cycle timing and phase transition [20,74,75], which contributes to cell transformation [76,77]. The proximity labelling and kinomics data suggested that, compared to Tax1a, Tax1b could exert a specific control over cell cycle-related pathways. This was validated by functional assays, demonstrating a more efficient blockade in G2/M phases by Tax1b than by other Tax1 proteins. G2/M blockade was previously used as a proxy for Tax1a oncogenic potential [78,79], and indeed, in our experimental setting, the efficient blockade in G2/M phases by Tax1b correlated with a higher transforming capacity of Tax1b compared to Tax1a. The increased Tax1b-induced blockade in G2/M did not seem to be caused by Tax1b-specific increase in double strand breaks induction, but could be due to a Tax1b-specific induction of other types of DNA damage, or to more efficient cell cycle checkpoint activation. Additional experiments will help determine the basis of Tax1b-specific features. Altogether, our data indicate that Tax1b has a slightly higher transforming potential than Tax-1a, while Tax-1c have lower transforming potentials and induce higher inflammatory signatures.

Although these conclusions seem to mirror reported differences in pathogenicity of HTLV-1a and HTLV-1c genotypes (there is no information regarding HTLV-1b), they should be regarded with caution as we performed most of our analyses on Jurkat T-cells transiently expressing Tax1 variants. While this is a useful experimental system to provide comprehensive insights in the functional properties of Tax1 by omics approaches, the transformed status of this cell line, with accumulated mutations and dysfunctions in PI3K signalling for instance, as well as high levels of genomic instability, might decrease the sensitivity of detection of Tax1-mediated cell reprogramming. Confirming these results in primary T-cells, or in animal models such as Tax transgenic drosophila [80] or Tax transgenic mice [81,82], would be an interesting follow-up of this work. Further insights into the functional comparison of HTLV-1 genotypes could also come from considering (i) the possibly genotype-dependent regulation of Tax expression through specific LTR activity or through specific post-transcriptional mechanisms, (ii) the implication of other viral determinants such as HBZ or auxiliary proteins that are involved in HTLV-1 proviral load, a predictive marker of pathogenesis [83,84], and that may be significantly different between genotypes [85–87]; (iii) the interplay between Tax and these other viral factors. We speculate that functional divergence among these other viral determinants could synergistically affect the fate of infected cells. Some recent studies have compared HTLV-1a and HTLV-1c properties upon infection with full viruses (including chimeric viruses), either in samples from people infected by HTLV-1, or in humanized mouse or macaque models [10,11], bringing strong evidence that both genotypes do indeed lead to distinct host/pathogen interactions *in vivo*, with an increased host inflammatory response that is consistent with our findings. We believe that combining these *in vivo* approaches with a reductionist strategy such as ours can prove highly informative. Lastly, our study focuses on representative sequences of three HTLV-1 genotypes, but do not span the entire phylogenetic diversity of these genotypes (we did not cover HTLV-1a-TC, -NA and -WA for instance), nor include other minor African genotypes such as HTLV-1d-g [12]. Extending comparative analysis to other strains or other genotypes should be considered in the future.

As a conclusion, we demonstrated that variations in Tax1 sequences among the three major HTLV-1 genotypes significantly influenced the reprogramming of Tax-expressing T-cells, with possible pathophysiological consequences. Efforts to better take the viral genetic variability into account are therefore encouraged to come up with relevant recommendations for people living with neglected or minor genotypes of HTLV-1.

## Materials and methods

### Constructs

Sequences were analysed using Geneious Prime (version 2021.0.3 and following). Tax1 variant sequences were synthetized by Genscript Biotech (Leiden, Netherlands) with codon optimization (OptimumGene algorithm). All optimized sequences show ≥ 69% of codons in the 90–100 quality group. Tax1 optimized sequences were cloned under the control of a CMV promoter into the pcDNA3.1(-)-myc-6xHis A backbone (referred to as pCMV-myc-6xHis), using the EcoRI and HindIII sites. Plasmids were amplified in *E. coli*, purified using NucleoBond Xtra Maxi kit (Macherey-Nagel Cat. No. 740414), and verified by Sanger sequencing using the T7 and BGHrev sequencing primers. pCMV-Tax1b-SF26/P11L-myc-6xHis plasmid (expressing the functional Tax1b-SF26 protein) was produced using the QuikChange II Site-Directed Mutagenesis Kit (Agilent, Cat. No. 200523) using the following primers: forward 5’-CCCTGGCTTTGGCCAGTCTCCCCTGTTCGGCTACCCC-3’ and reverse 5’ CACGGGGTAGCCGAACAGGGGAGACTGGCCAAAGCCAGGG-3’. pEF vectors encoding Tax1-myc variants were generated by cloning Tax1 sequences from the pCMV-Tax1-myc-6xHis vectors into the pEF-myc-cyto vector between the XhoI and NcoI sites. BioID2 constructs expressing myc-BirA*-Tax1 variants were generated by cloning Tax1 sequences from the pCMV-Tax1-myc-6xHis vectors into the myc-BioID2-MCS vector (Addgene #74223, from Dr Kyle Roux), between the EcoRI and HindIII sites. Additional plasmids used in this study are the following: pMACS-LNGFR plasmid (Miltenyi, Cat. No. 130-091-890); HTLV-1-LTR-luciferase and NF-κB-luciferase reporter plasmids [61], Renilla luciferase reporter vector (phRG-TK, Promega, Cat. No. E6921).

### Phylogenetic analysis

*Tax* sequences were extracted from 26 complete HTLV-1 genomes available in GenBank. Accession numbers for the sequences of interest are the following: HTLV-1a_ATK: J02029; HTLV-1b_SF26: JX507077; HTLV-1c-mel_EM5: KX905202; HTLV-1c-aus_AusCS: KF242506. Sequence alignment was performed with MUSCLE [88] under the SeaView interface (version 5.0.4). Phylogenetic trees were constructed in SeaView using the maximum likelihood (PhyML) method and validated by parsimony analysis. The robustness of the resulting clades was assessed using the approximate likelihood-ratio test (aLRT).

### Cell culture

Jurkat T-cells were maintained at 37°C with 5% CO_2_ in RPMI 1640 Medium (1X) + GlutaMAX-I (Gibco Cat. No. 61870044) supplemented with Fetal Bovine Serum (FBS, 10%) and Penicillin/Streptomycin (100 U/ml Gibco Cat. No. 15140122). HEK293T and NIH Rat-1 208Fp24 were maintained at 37°C with 5% CO_2_ in DMEM, high glucose, GlutaMax Medium (1X) supplemented with FBS (10%) and Penicillin/Streptomycin. Microporation of Jurkat with plasmids expressing Tax variants was performed using Neon Transfection System (Invitrogen Cat. No. MPK10096). Cells were washed in PBS, concentrated at 2 millions/ 100 µL in resuspension buffer and incubated at a ratio of 10 or 20 µg DNA for 2 million cells depending on the plasmid. The microporation program was the following: 1350V voltage, 10 ms pulsation time, 3 pulsation repetition. After microporation, cells were resuspended in RPMI 1640 + GlutaMAX-I supplemented with FBS (10%) for 20h. For microporation of Jurkat T-cells prior to enrichment of Tax-expressing cells, cells were co-microporated with a DNA mix containing pMACS-LNGFR plasmid and Tax-expressing plasmids as described above at a ratio 1:20. NIH Rat-1 208Fp24 were transfected with pCMV-Tax-myc-6xHis plasmids using Lipofectamine 2000 (Invitrogen, ref. 11668–019) according to manufacturer’s instructions.

### Magnetic enrichment of Tax-expressing cells

LNGFR^+^ Jurkat T-cells were sorted using MACSelect LNGFR System (Miltenyi Cat. No. 130-091-330). After washing steps and incubation with MACSelect LNGFR MicroBeads (Miltenyi. Cat. No. 130-091-330) following manufacturer’s instructions, cells were sorted on autoMACS Pro Separator (Miltenyi, Cat. No. 130-092-545) using the Posseld program, as specified in the instructions. One tenth of the positive fraction was used for cytometry staining, and the remaining positive cells were centrifuged and stored as a dry pellet at -80°C until RNA or protein extraction.

### Western blot and ubiquitination assay

After microporation, cells were harvested and washed in PBS. For classical SDS-PAGE analysis, cells were lysed on ice for 20 min in RIPA (50 mM Tris pH 7.4, 150 mM NaCl, 1% NP40, 0.25% NaDOC), complemented with protease inhibitors (Complete, Roche) and 1 mM phenylmethylsulfonyl fluoride (PMSF). After centrifugation 15,000 rpm, 10 min, 4°C, supernatants were collected and protein concentration determined using Bradford (Biorad). Ubiquitination assay was performed using a denaturing Ni-NTA pulldown procedure described previously [62]. For SDS-PAGE analysis, proteins were separated on 4–12% Bis-Tris NuPAGE gels (Thermo Fisher Scientific, ref. NP0336BOX, NP0335BOX) and transferred onto PVDF membranes (Immobilon P, Millipore, ref. IPVH00010). Membranes were saturated in TBS-Tween (Sigma-Aldrich, ref. P1379) containing 5% milk. Membranes were then incubated in indicated primary antibodies for 1h at room temperature (RT), or over-night (ON) at 4°C, washed 3 x 5 min with TBST and then incubated with secondary antibodies for 1h at RT, or ON at 4°C. Revelation was performed using ECL Prime (Amersham, ref. RPN2232). The following antibodies were used: mouse anti-myc (4A6, Millipore, ref. 05–724), mouse anti-tubulin (Sigma, ref. T6074 or T9026), mouse anti-ubiquitin (Santa Cruz, ref. sc-8017), mouse anti-Chk1 (Sigma, ref. C9358), rabbit anti-phospho-Chk1 (Ser296) (Cell Signaling Technologies, ref. 2349), secondary anti-mouse-HRP (Sigma, ref. A9044) and anti-rabbit-HRP (Santa Cruz, ref. sc-2357).

### Luciferase assays

Jurkat cells were co-microporated with Tax-encoding vectors, together with the HTLV-1-LTR-luciferase or the NF-κB-luciferase reporter vectors and a Renilla luciferase reporter vector (phRG-TK) used as a microporation efficiency control. Cells were lysed 20h after transfection and luciferase activity was measured using the Dual-Luciferase Reporter Assay System (Promega, Cat. No. E1960), according to the manufacturer’s instructions, on a Mithras plate reader luminometer (Berthold).

### Confocal analysis

For microscopy samples preparation, 1.5 High Precision glass coverslips (Thermo Fisher Scientific, Cat. No. 11846933) were coated using 500 µL of poly-L-lysine (Sigma-Aldrich, Cat. NO. P4832-50ML) for 5 min and rinsed with sterile H_2_O. After drying, cells were plated (≈ 500 000 cells per coverslip) for 30 min at RT before fixation using Paraformaldehyde (PFA, Electron Microscopy Science, Cat. No. 50-980-493) 4%, 20 min at RT. Coverslips with cells were washed in PBS x3, treated with NH_4_Cl for 10 min, RT, washed in PBS x3 and permeabilized using PBS-Triton X100 0.5% for 10 min, RT. Cells were then saturated using PBS Tween 0.2% (PBST) with BSA 5% during 30 min RT then stained in PBST BSA 5% for 45 min, RT and mounted in microscopy slides using ProLong Diamond Antifade Mountant with DAPI (Invitrogen, P36962) or stained with Hoechst and mounted using ProLong Diamond Antifade Mountant (Invitrogen, P36961). Microscopy images were obtained using an inverted Zeiss Axio Observer Z1 LSM800 confocal microscope, equipped with a 63x Oil 1.4 DIC M27 oil immersion objective under the Zen software. Images were processed with Fiji [89]. The following antibodies were used: goat anti-His (Bethyl Lab, ref. A190-113A), mouse anti-GM130 (BD, ref. 610822), mouse anti-myc (4A6, Millipore, ref. 05–724), rabbit anti-GM130 (Abcam, ref. ab52649), rabbit anti-p65 (Santa Cruz, sc-372), secondary anti-goat AF488 (Abcam, ref. 150129), anti-mouse AF647 (Abcam, ref. ab150107), anti-mouse DyLight488 (Vector, ref. DI-2488), anti-rabbit AF568 (Abcam, ref. ab175693).

### Flow cytometry and image cytometry

For flow cytometry and image cytometry, cells were harvested in conic Greiner CELLSTAR 96 well plates (Sigma-Aldrich, ref. M0812), centrifuged at 2000 rpm, 2 min and washed in PBS containing 2mM EDTA (PBS-EDTA). Cells were stained using eBioscience Fixable Viability Dye eFluor 780 (Invitrogen, ref. 65-0865-14) for 20 min, 4°C, obscurity. After wash in PBS-EDTA, cells were fixed in PFA 4% prepared in PBS-EDTA for 20 min, RT, and permeabilized and saturated in PBS containing 0.1% Triton X100 and 2% BSA (Sigma-Aldrich, ref. A7906) for 5 min. Cells were the incubated in PBS-EDTA containing 5% BSA for more than 30 min before staining. Tax-myc proteins were stained with a mouse anti-myc-FITC antibody (Sigma, ref. F2047) prepared in PBS-EDTA with 2% BSA. For flow cytometry, samples were acquired on Canto II (BD Biosciences). Analyses of cytometry results were performed using FlowJo (v.10 and later).

For image cytometry, cells were also stained with a rabbit anti-GM130 antibody (Abcam, ref. ab52649) followed by an anti-rabbit AF568 secondary antibody (Abcam, ref. ab175693), and counterstained with Hoechst. Acquisition of image cytometry data was performed using ImageStreamX Mark II Imaging Flow Cytometer (Cytek, Amnis), with a 4 lasers instrument (405nm, 488nm, 561nm and 642nm). Images were collected within INSPIRE software at X60 magnification, low flow rate with activated Extended Depth of Field (EDF) to improve detection of the Golgi apparatus in focus. The following channels were used: Channel 1 (brightfield), Channel 2 (FITC, Tax-myc), Channel 4 (AF568, GM130), Channel 6 (SSC), Channel 7 (Hoechst), Channel 9 (brightfield 2), Channel 12 (APC-Cy7, Live/Dead). Images from ImageStreamX MarkII were collected and analysed using the IDEAS (v.6.0) software. The minimum number of Tax-myc-positive cells collected per variant and per replicate was approx. 3000 cells. The mask for the “Golgi” compartment was defined based on a threshold value on Channel 4 (AF568, GM130) fluorescence signal. The detected area was then dilated of 1 pixel. The mask for the “Nucleus” compartment was defined based on a threshold value on Channel 7 (Hoechst) signal. The detected area was eroded of 2 pixels to compensate the propensity of Hoechst signal to spread, and the area included in the “Golgi” mask was subtracted to ensure the exclusive attribution of pixels to a single given mask. The mask for the “Cytoplasm” compartment was defined as the Object (automatic detection of cells based on brightfield channels in the IDEAS software) dilated of 1 pixel, after subtraction of the areas included in the “Golgi” or “Nucleus” masks. The list of image-based variables (referred to as “features”) extracted from IDEAS is provided in S4 Table. All variables were centered and reduced before being analyzed with the UMAP (Uniform Manifold Approximation and Projection) dimension reduction algorithm in R (n_neighbors = 5, min_dist = 0.05, n_components = 2). Note that the “Object Number” and “Variant” features were excluded for the UMAP calculation.

### BioID procedure and mass spectrometry analyses

Jurkat T-cells were microporated with myc-BirA*-Tax constructs with a DNA ratio of 10 µg DNA for 2 million cells as described in microporation section. After microporation, cells were resuspended in RPMI 1640 + GlutaMAX-I supplemented with FBS (10%) for 20h with addition of 50 µM Biotin (Sigma-Aldrich, Cat. No. B4501). After 20h, cells were washed in PBS and processed as previously described [90]. Briefly, streptavidin pulldown was performed on whole cell lysates using Streptavidin C1 Dynabeads MyOne (Invitrogen, Cat. No. 65002), on wheel, ON at 4°C. After the washing steps, samples were resuspended quickly in Urea 3M in Ammonium Bicarbonate 50 mM to eliminate detergents and then washed twice in Ammonium Bicarbonate 50 mM. After the last washing step, samples were resuspended and digested in 50 µL PreOmics Lysis Buffer with Trypsin/LysC mixture for 3h at 37°C and then desalted following the manufacturer protocol (iST kit ref. P.O.0001, PreOmics). Peptide samples were dried and resuspended in Formic Acid (FA) 0.1% and quantified using the Pierce Quantitative Peptide assay (Thermofisher Scientifics, ref 23290). Finally, 400 ng of each sample were injected in the mass spectrometer.

Samples were analyzed in a Label Free quantitation strategy using an Ultimate 3000 nano-RSLC (Thermo Scientific, San Jose California) coupled on line with a Q Exactive HF mass spectrometer via a nano-electrospray ionization source (Thermo Scientific, San Jose California). Samples were injected and loaded on a PepMap NEO C18 trap-column 300 μm ID x 5 mm, 5 μm, (Thermo Scientific) for 3.0 minutes at 20 μL/min with 2% ACN, 0.05% TFA in H_2_O and then separated on a C18 Acclaim Pepmap100 nano-column, 50 cm × 75 μm i.d, 2 μm, 100 Å (Thermo Scientific) with a 50-minutes linear gradient from 3.2% to 20% buffer B (A: 0.1% FA in H2O, B: 100% ACN, 0.1% FA) and from 20% to 40% of B in 10 min and then from 40 to 90% of B in 2 min, hold for 10 min and returned to the initial conditions in 1 min for 14 min. The total duration was set to 90 minutes at a flow rate of 300 nL/min. The oven temperature was kept constant at 40 °C. Samples were analysed with TOP15 DDA HCD method: MS data were acquired in a data-dependent strategy selecting the fragmentation events based on the 15 most abundant precursor ions in the survey scan (350–1600 Th). The resolution of the survey scan was 120,000 at m/z 200 Th and for MS/MS scan the resolution was set to 15,000 at m/z 200 Th. The Ion Target Value for the survey scans in the Orbitrap and the MS/MS scan were set to 3E6 and 1E5 respectively and the maximum injection time was set to 60 ms for MS and MS/MS scan. Parameters for acquiring HCD MS/MS spectra were as follows: collision energy = 27 and an isolation width of 1.4 m/z. The precursors with unknown charge state, charge state of 1 and 8 or greater than 8 were excluded. Peptides selected for MS/MS acquisition were then placed on an exclusion list for 20 s using the dynamic exclusion mode to limit duplicate spectra. Data files were then analyzed with Proteome Discover 2.5 using the SEQUEST HT algorithm against the Swissprot Human database (2022-07 release, 20316 sequences), complemented with the Uniprot HTLV sequences (2023-06 release, 9 sequences), the BirA and streptavidin sequences, Tax1 variants sequences and common contaminants. The fasta files of the databases used have been deposited together with the dataset via the MassIVE tool (dataset identifier MassIVE MSV000100656). Precursor mass tolerance was set at 10 ppm and fragment mass tolerance was set at 0.02 Da, and up to 2 missed cleavages were allowed. Oxidation (M), biotin (K), acetylation (Protein N-terminus) were set as variable modification, and Carbamidomethylation (C) as fixed modification. Peptides and proteins were filtered with a false discovery rate (FDR) at 1% using percolator. Protein quantification was done by the Label Free Quantification (LFQ) approach, and abundance values were obtained for each sample, normalized to the total peptide amount. The abundance ratios [BirA*-Tax1/ BirA*] and [BirA*-Tax1x/ BirA-Tax1a*] were calculated in a pairwise way. Statistical validation was based on t-test and a protein is considered differentially abundant between two conditions if the abundance ratio (referred to as FoldChange in the text) is ≥ 1.5 or ≤ 0.67 (|log2FoldChange| threshold of 0.58) with a p-value ≤ 0.05. For proteins present in one condition but absent in the other, the abundance ratio was artificially set at 100 or -100 (|log2FoldChange| = 6.64) and the p-value at 10^-17^.

### BioID analysis and over-representation analysis

Analysis of BioID results was made in R (v.4.3.2). All volcano plot and representation were made using ggplot2 (v.3.5.1) R package. Over-representation analyses (ORA) were performed using clusterProfiler R package [91] with a |log2FoldChange| threshold of 0.58 and with a p-value cutoff of 0.05. To allow analysis against multiple databases, Uniprot ID provided by mass spectrometry analysis were attributed to Ensembl genes ID, entrez gene ID or hgnc ID using biomaRt package (v.2.58.2) [92].

### RNA extraction

RNA extraction was performed on Tax-expressing cells magnetically enriched 20 hours post-microporation, using the NucleoSpin RNA kit (Macherey-Nagel, Cat. No. 740955.50). RNA was eluted in 60 μl RNase-free H_2_O, and concentration and absorbance ratios were checked by Nanodrop 2000 (Thermo Scientific Cat. No. ND-2000) before storage at -80°C until sequencing.

### Bulk RNAseq library and sequencing

Library preparation and sequencing were performed by Novogene with poly-A enrichment. Sequencing was performed using Illumina Sequencing PE150. Sequencing was performed in paired-end, resulting in the collection of 2 sequencing files per sample.

### Processing of data and pseudo-alignment

Prior to alignment, sequencing data were submitted to quality check using fastQC ([93], v.0.11.8), trimmed using FastP ([94], v.0.23.2) to eliminate short or low-quality reads and adaptors. Pseudo-alignment of reads was made on human transcriptome GRCh38 obtained from Ensembl database (GRCh38.111 version) using SALMON ([95], v.1.10.1). For quality check of samples, Tax1 variants transcripts were manually added as an additional chromosome.

### Differential expression analysis

Count tables were generated from SALMON pseudo-alignment files using *tximport* [96] and processed in R (v.4.3.2) using the DESeq2 package (v.1.42.1) for normalization of counts [97]. After quality check from normalized counts, only cellular genes were kept for further analysis. Differentially Expressed Genes (DEG) analysis was performed between 2 conditions following by log2FoldChanges after shrinkage using apeglm (v.1.24.0) [98]. Significant DEGs were selected using a |log2FoldChange| threshold of 0.58 (|FoldChange| threshold 1.5) and an adjusted p-value cutoff of 0.05 (95% confidence).

### Over-representation analyses

Similar to BioID analysis, ORA were performed using the clusterProfiler package for R (v.4.10.1), using the list of genes present in the count table as the background list. According to the database tested (KEGG, Gene Ontology or Reactome) [99,100], Ensembl genes ID generated during the previous step were associated to entrez gene ID or hgnc ID using biomaRt package (v.2.58.2) [92]. For Gene Ontology analysis, enrichGO function from clusterProfiler was used against org.Hs.e.g.,db database. For all ORA, the p-values were adjusted using Benjamini-Hochberg procedure to calculate the False Discovery Rate (FDR). The adjusted p-value cutoff used was 0.05 and the |log2FoldChange| threshold 0.58.

### Kinomics procedure and analysis

Kinomics experiments were performed using the Pamgene technology, following the manufacturer’s instructions. Triplicates of enriched Tax1-expressing cell samples were lysed in the provided lysis buffer, prepared according to the manufacturer’s information and analysed on the PamStation12 for PamChip analysis. Each replicate was prepared in duplicate for both PTK PamChip and STK PamChip processing. Images of each array were taken at several exposure times by a camera in the workstation and later used by the BioNavigator software to calculate signal values for each phosphosite. Upstream Kinase Analysis (UKA) algorithm was used by the Pamgene Science team to predict differential kinase activity from phosphorylated phosphosite abundance, using knowledge from publicly available databases of kinase-substrate relationships. By default, all kinome comparison was made against the Tax1a reference condition.

### Lentiviral production and transduction

HEK293T cells were transfected with psPAX2 (encoding HIV Gag/Pol, 4.68 μg; Addgene #12260), pMD2.G (vesicular stomatitis virus G protein [VSV-G], 2.52 μg; Addgene #12259) and pGenLenti-Tax1-myc (Tax from the different genotypes, 9 μg; Genscript) plasmids. After 72 h, supernatants were collected, centrifuged, and stored at −80°C. For Tax transduction, 0.3 million Jurkat T-cells were incubated with lentiviruses (500µL of supernatants from lentiviral production) together with polybrene (8µg/mL, Invitrogen) for 1 h. After spinoculation at 800g for 45min, cell pellets were resuspended, and cells were cultured for 48h.

### Cell cycle and genotoxicity

At 48h post-transduction, Jurkat T-cells were stained with LIVE/DEAD fixable green (Invitrogen) for 15 min and fixed in PFA 4% for 20 min. After permeabilization for 10 min in PBS 0.1% BSA and 0.1% Triton X-100, cells were stained for 30 min at RT with anti-γH2AX IgG mouse (Cell Signaling Technologies, Cat. No. 803125) and anti-Myc IgG rabbit (Invitrogen, Cat. No. PA1981) antibodies as well as Vybrant DyeCycle Ruby Stain (Invitrogen, Cat. No. V10309). After washing with PBS 0.1% BSA, cells were stained with anti-rabbit BV421 (BD bioscience, Cat. No. 565014) and anti-mousee AF488 (Invitrogen, Cat. No. A11001) secondary antibodies for 30 min without light. For flow cytometry, samples were acquired on Cytoflex (Beckman). Analyses of cytometry results were performed using FlowJo (v.10 and later).

### Proliferation assay

Jurkat T-cells were stained with CellTrace Far Red (Invitrogen, Cat. No. C34564) in PBS for 20 min and washed in Jurkat medium for 2h. After 24h of incubation, cells were transduced, and fixed 48h after transduction. Fixed cells were stained with anti-Myc IgG rabbit (Invitrogen, Cat. No. PA1981) and anti-rabbit BV421 (BD bioscience, Cat. No. 565014) antibodies, and analyzed by flow cytometry. Proliferation rates were assessed by measuring the MFI of the CellTrace signal.

### Soft agar colony formation assay

After 20h, transfected NIH Rat1 cells were detached using Trypsin-EDTA and resuspended in DMEM + FBS + P/S. Transformation assays were performed using CytoSelect 96-well Cell Transformation Assay kit (Cell Biolabs, Cat. No. CB-CBA-130) in 96-w plates and maintained for 6–8 days. Division clusters were quantified by screening each well using optic microscope and colony number and size were measured using ImageJ.

### Statistical analyses

Flow cytometry data were analyzed using FlowJo v10 software (Tristar). GraphPad Prism 9 and 10 were used to generate figures and for statistical analysis. Statistical significance between different conditions was calculated using the tests indicated in the corresponding figure legends.

## Supporting information

S1 Fig Expression of Tax1 variants constructs in several cell types and enrichment of Tax-expressing cells (related to Fig 1). **A.** Alignment of the 328–340 amino-acid stretch among Tax1a and Tax1c sequences. **B. Left:** Alignment of the 1–12 amino-acid sequence among Tax1b sequences. **Right:** Jurkat T-cells were microporated with the pCMV-Tax1-myc-6xHis constructs or an empty control and cultured for 20h before analysis by western blot using an anti-myc antibody. Tubulin was used as a loading control. Here, “Tax1b” refers to the published Tax1b-SF26 sequence. **C.** Gating strategy of Jurkat T-cells microporated with pEF-Tax1-myc constructs for flow cytometry analysis. After gating cells on FSC/SSC and single cells on FSC-A/FSC-H, live cells were gated on the live/dead (LD) channel. Tax-expressing cells were gated on anti-myc-FITC channel against empty control. **D.** Jurkat T-cells were microporated with the pCMV-Tax1-myc-6xHis constructs and cultured for 20h before analysis by western blot (left) or flow cytometry (right) using anti-myc antibodies. The percentage of Tax-myc-positive cells and the mean fluorescence intensity (MFI) were determined. The histogram shows a representative replicate (dotted line represents Tax^+^ gate, and percentages of Tax^+^ cells and MFI of this replicate are indicated in italic), and values of percentage of positive cells for independent replicates are shown on the right bar chart. Plots show individual values and medians (n = 6). **E.** NIH Rat-1 cells were transfected with the pCMV-Tax1-myc-6xHis constructs and cultured for 20h before analysis by western blot (left) or flow cytometry (right) using anti-myc antibodies. The percentage of Tax-myc positive cells and the MFI were determined. The histogram shows a representative replicate (dotted line represents Tax^+^ gate, and percentages of Tax^+^ cells of this replicate are indicated in italic), and values of percentage of positive cells for independent replicates are shown on the right bar chart. Plots show individual values and medians (n = 7). **F.** A sorting procedure based on co-transfection of LNGFR (membrane peptide form) was used to enrich Tax-expressing cells. (Left) Schematic representation of the procedure. Created in BioRender. Dutartre, H. (2026) https://BioRender.com/z7n6kuw. (Right) Jurkat cells were co-microporated with pEF-Tax1-myc constructs and pMACS-LNGFR and cultured for 20h before positive sorting with anti-LNGFR magnetic beads. Pre- and post-sorting percentages of Tax-positive cells were assessed by flow cytometry using an anti-myc-FITC antibody (n = 5).
(TIF)

S2 Fig Image cytometry-based quantification of Tax1 subcellular distribution (related to Fig 2). **A**. Amino acid sequence alignment of the 1–60 and 180–202 regions of Tax1 variants, encompassing the nuclear localisation sequence (NLS) and nuclear export sequence (NES) of Tax1, respectively. Key residues in the NES are boxed. Clustal X colour scheme is used to highlight the chemical properties of amino acids (hydrophobic [V, I, L, M]: green; aromatic [F, Y, W]: blue; positive charge [H, R, K]: red; polar [S, T], glycine and proline: orange). **B**. UMAP replicates of image cytometry analysis as represented using FlowJo v10 software. Cells were ranked according to the relative contribution of Tax1 signal in the Golgi (left), nucleus (middle) or cytoplasm (right) masks, and ranks were displayed as colour ranging from blue to red. Cell subpopulations showing a high contribution of Tax1 signal in each of these masks were gated, with the median rank of each subpopulation kept equivalent among replicates. **C**. Individual UMAPs obtained for each replicate and deconvolved according to the variant of interest. Cell frequencies in each gated subpopulation were retrieved and used for statistical analysis.
(TIF)

S3 Fig Localisation and biotinylation capacity of BirA*-Tax1 constructs in Jurkat cells (related to Fig 2). **A**. Jurkat T-cells were microporated with the pCMV-myc-BirA*-Tax1 constructs and cultured for 20h before analysis by immunofluorescence using anti-myc (yellow) and anti-GM130 (magenta) antibodies. The right panel shows an enlarged view of the Golgi area (scale bar = 10 µm). **B**. Jurkat T-cells were microporated with the pCMV-myc-BirA*-Tax1 constructs or empty control and cultured in the presence of 50 µM Biotin for 20h before analysis by western blot using streptavidin-HRP. **C.** Venn diagram of enriched or depleted proteins from individual “Tax1x vs Tax1a” comparisons. The number of proteins in each area and the corresponding percentage relative to the total protein number are indicated.
(TIF)

S4 Fig Validation of the RNA-Seq dataset generated in this study, compared to previous studies (relative to Fig 3). **A.** Up-regulated (left) and down-regulated (right) differentially expressed genes (DEGs) identified in the “Tax1a vs empty control” comparison of the current dataset were compared to previously published transcriptomics data from Tax1a-expressing or HTLV-1-infected cells [30,43,44]. **B.** Volcano plot of DEGs (|log_2_FC| ≥ 0.58 and adj. p-value ≤ 0.05) identified in the “Tax1a vs empty control” comparison. Examples of up-regulated genes consistent with previous studies are highlighted in red, including NF-κB target genes (NFKB1, Rel, ICAM-1), JUN/FOS target genes, and cytokines (IL-13). **C.** Enrichment analysis of DEGs identified as up-regulated in the “Tax1a vs empty control” comparison. Enrichment analysis was performed across the KEGG database. GeneRatio corresponds to the ratio between the number of genes enriched in specific term and the total number of input genes.
(TIF)

S5 Fig Transcriptomics analysis of Tax1c-mel-expressing T-Jurkat cells compared to Tax1a. **A**. Scatter plot of the 261 DEGs in the “Tax1c-mel vs Tax1a” comparison. The scatter plot shows the fold changes in the “Tax1a vs empty control” and “Tax1c-mel vs empty control” comparisons, in the x- and y-axis, respectively, allowing classification of the genes into 5 categories, which proportions are represented on the right (see text for additional details). **B.** Enrichment analysis of DEGs in the “Tax1c-mel vs Tax1a” comparison. Enrichment analysis was performed across the KEGG database and the Reactome pathways. **C.** PROGENy inference of activation score of the indicated signalling pathways. Pathways inferred as more activated in Tax1a- or Tax1c-mel-expressing samples are depicted in orange or pink, respectively. **D.** Gene count heatmap of DEGs in the “Tax1c-mel vs Tax1a” comparison annotated as genes modulated downstream of the NF-κB pathway in the PROGENy database. Gene counts were normalized by rows.
(TIF)

S6 Fig Cell cycle modulation and cell transforming capacity of Tax1 variants (related to Fig 7). **A.** Jurkat T-cells were transduced with Tax1-myc expressing lentiviruses and cultured for 48h before analysis by flow cytometry using anti-myc antibodies. The percentage of Tax-myc positive cells and the mean fluorescence intensity (MFI) of the Tax-myc signal are shown for n = 6 independent replicates (mean with SEM). Statistical analysis: mixed-effect analysis with Tukey’s multiple comparisons test, *P < 0. 05. **B.** Jurkat T-cells were transduced with Tax1-myc expressing lentiviruses and cultured for 48h before analysis by flow cytometry using anti-γH2AX, anti-myc antibodies and DNA intercalant. The histogram shows a representative replicate (left). Mean fluorescence intensity (MFI) of γH2AX was quantified for 5 independent replicates after gating cells in the G1 or G2/M phase of the cell cycle, respectively (right). Data are mean with SEM. Statistical analysis: repeated-measures one-way ANOVA with Tukey’s multiple comparisons test, *P < 0. 05.
(TIF)

S1 Table Lists of enriched or depleted proteins identified in the BioID assay. (XLSX)

S2 Table Lists of DEGs identified in RNA-Seq. (XLSX)

S3 Table Lists of differentially active tyrosine kinases inferred from the kinomics assay. (XLSX)

S4 Table List of features extracted from IDEAS and used in the UMAP analysis of image cytometry data. Note that the “Object Number” and “Variant” features were excluded for the UMAP calculation.
(XLSX)

S1 Raw Images Original uncropped and unadjusted images underlying all blot results reported in the manuscript. (PDF)
