# Supplementary material for: A multi-omics approach reveals specific oncogenic and inflammatory potential of viral oncoproteins Tax from neglected HTLV-1b and -1c genotypes
Source: PLoS Pathog. 2026 Aug 25;22(8):e1014512. doi: 10.1371/journal.ppat.1014512 (PMC13537692; doi:10.1371/journal.ppat.1014512)
Supplement: Supplementary file 1 [file ppat.1014512.s011.pdf]

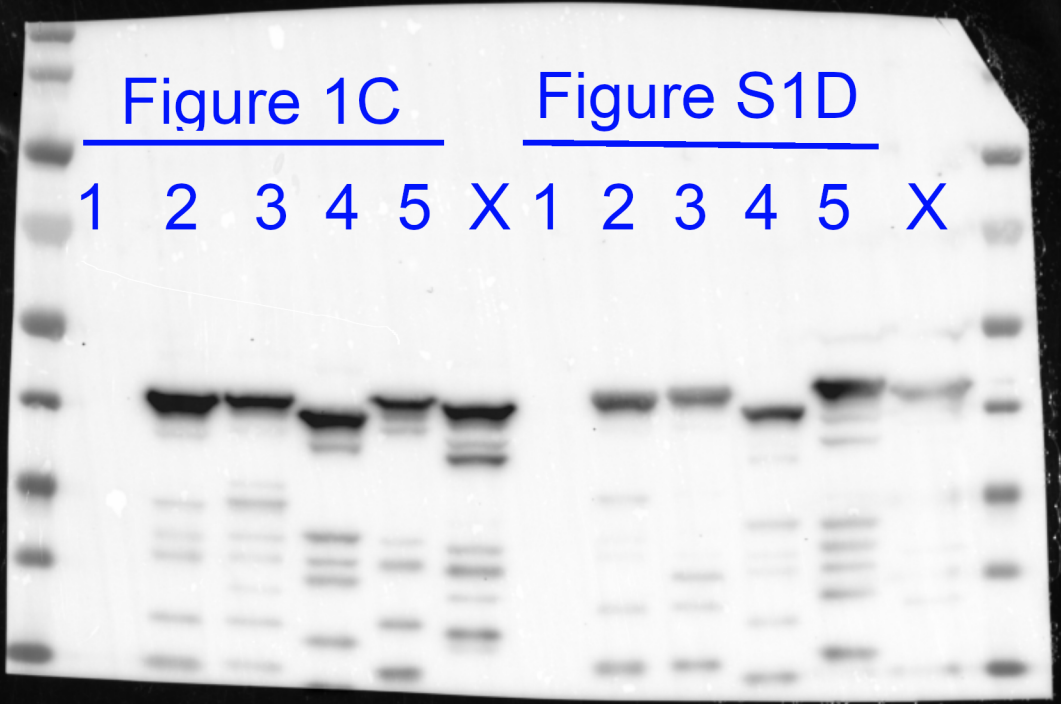

1: Empty vector
2: Tax1a
3: Tax1b
4: Tax1c-mel
5: Tax1c-aus

Anti-myc (Tax)

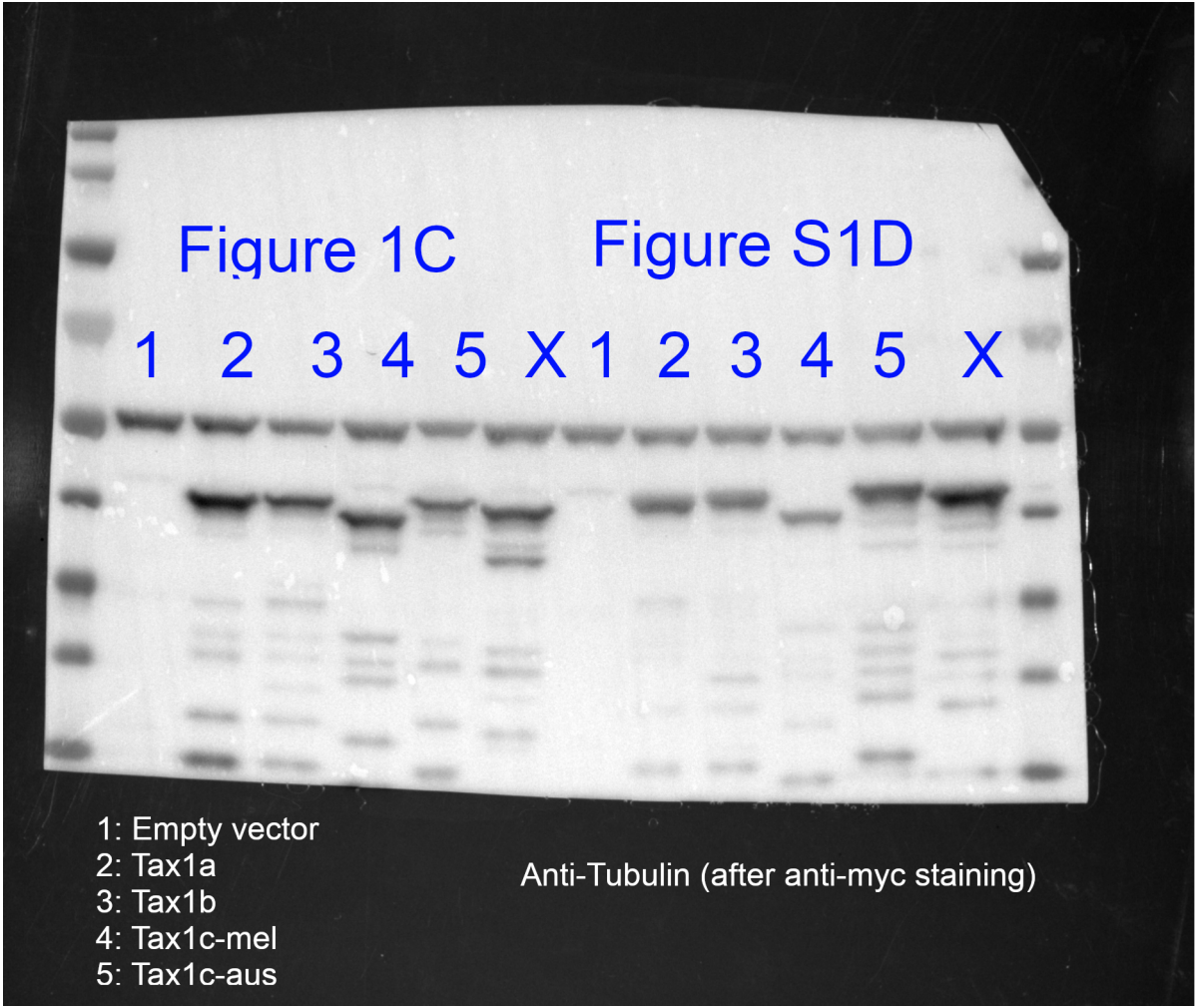
Figure 1C
Figure S1D
1 2 3 4 5 X 1 2 3 4 5 X
1: Empty vector
2: Tax1a
3: Tax1b
4: Tax1c-mel
5: Tax1c-aus
Anti-Tubulin (after anti-myc staining)

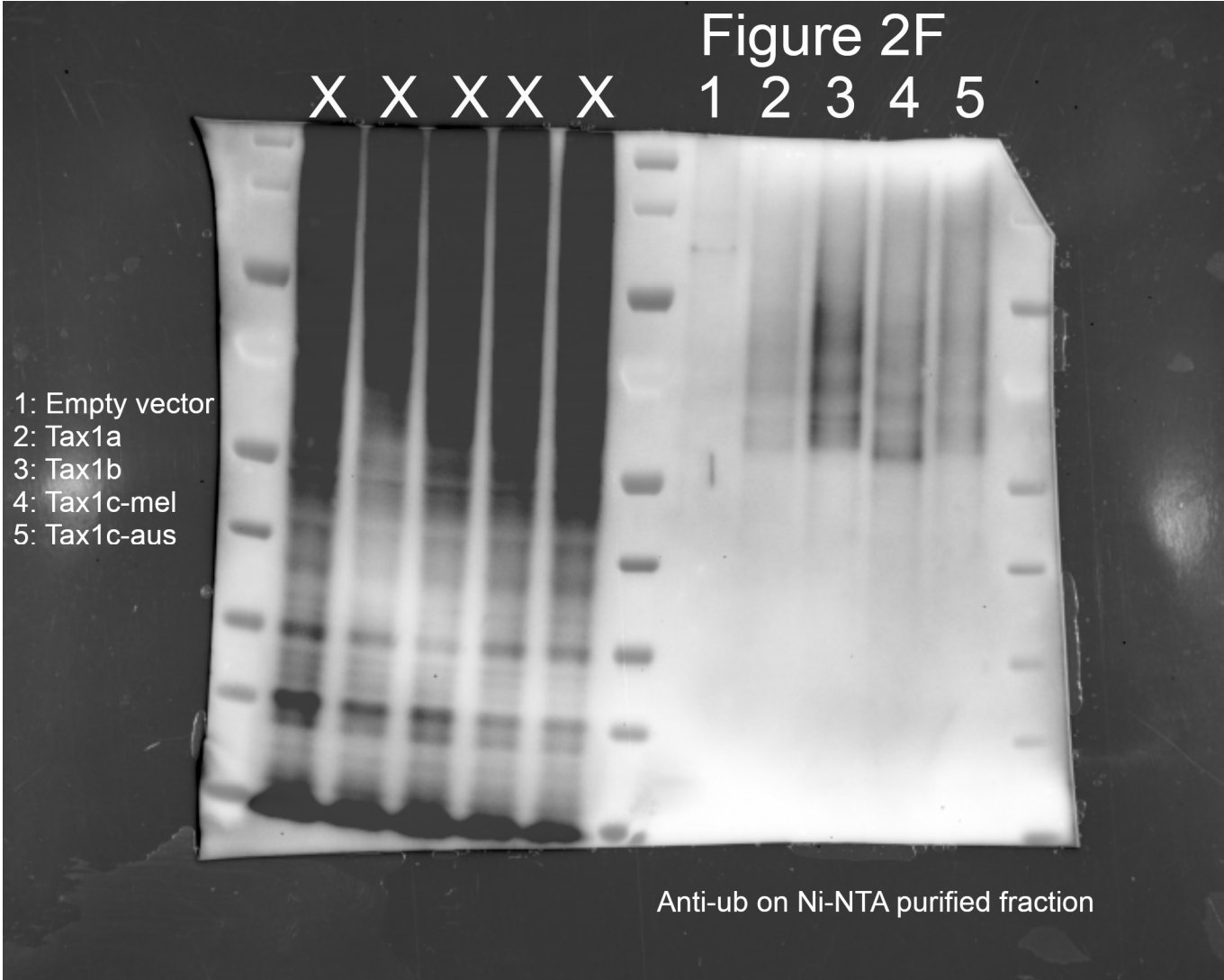
Figure 2F
X X X X X
1 2 3 4 5
1: Empty vector
2: Tax1a
3: Tax1b
4: Tax1c-mel
5: Tax1c-aus
Anti-ub on Ni-NTA purified fraction

Figure 2F

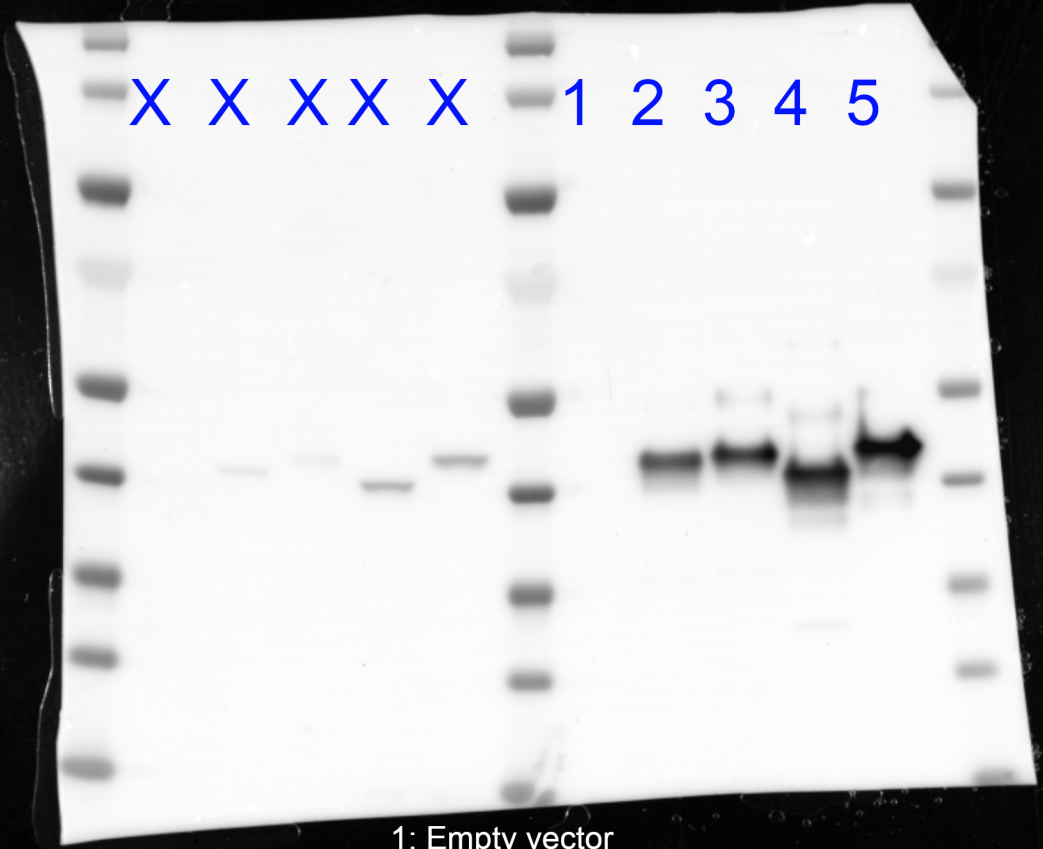

1: Empty vector
2: Tax1a
3: Tax1b
4: Tax1c-mel
5: Tax1c-aus

Anti-myc (Tax) on Ni-NTA purified fraction

Figure 2F

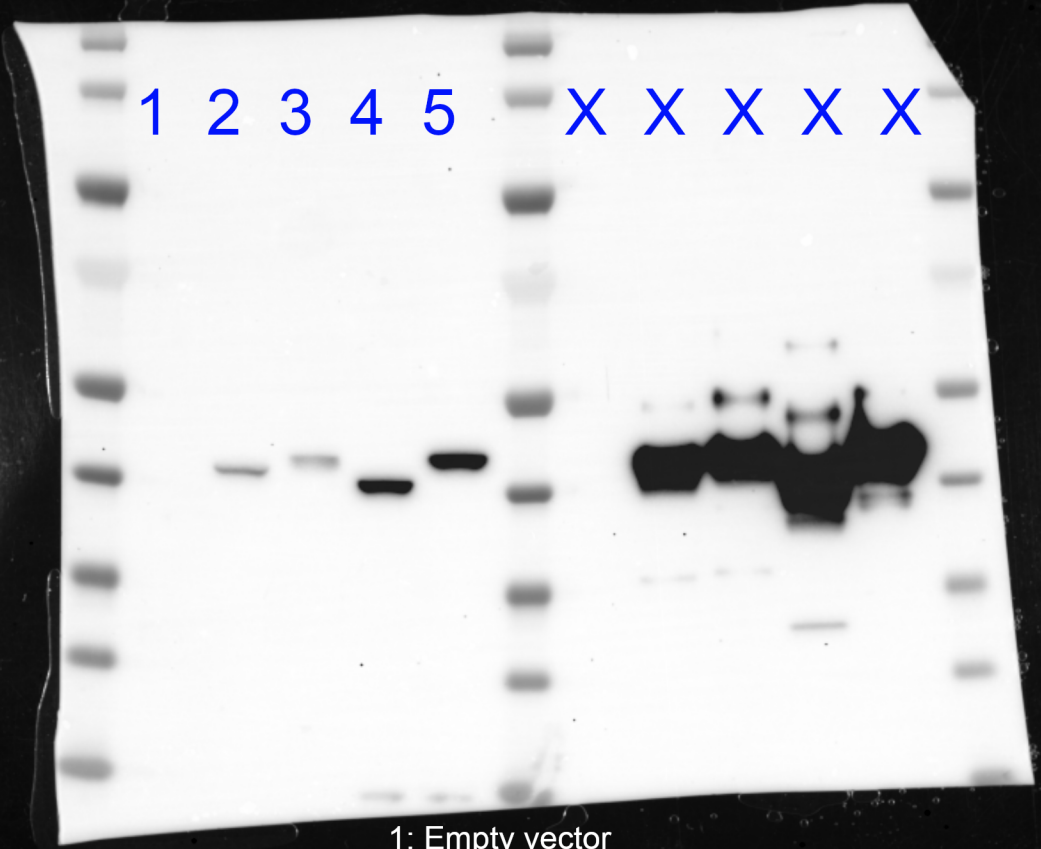

Anti-myc (Tax) on input

1: Empty vector
2: Tax1a
3: Tax1b
4: Tax1c-mel
5: Tax1c-aus

Figure 6E

Merge (with colorimetric)

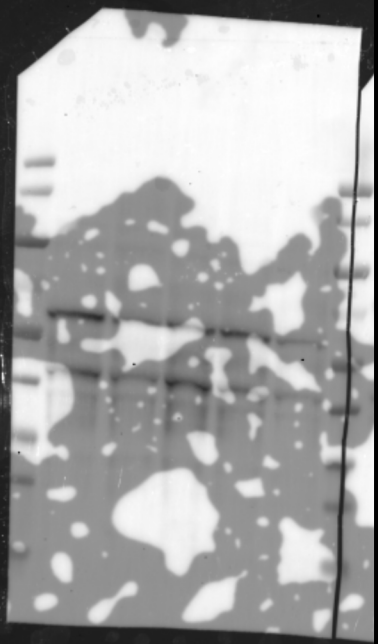

Anti-Phospho-Chk1 (S296)

Chemiluminescence only

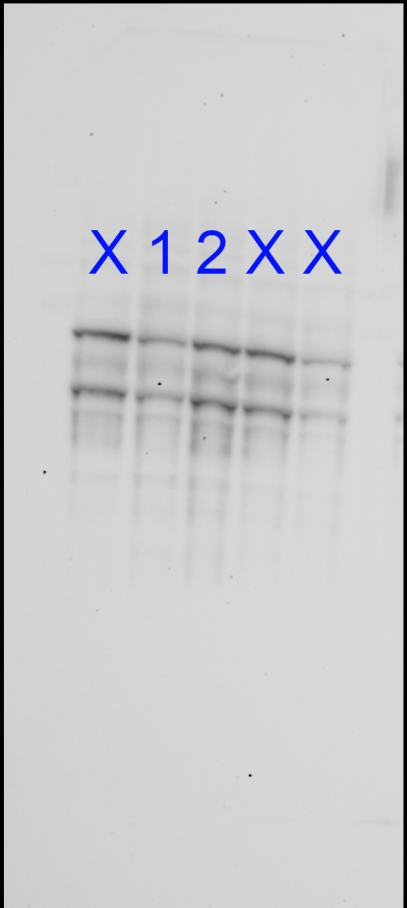

1: Tax1a
2: Tax1b

Figure 6E

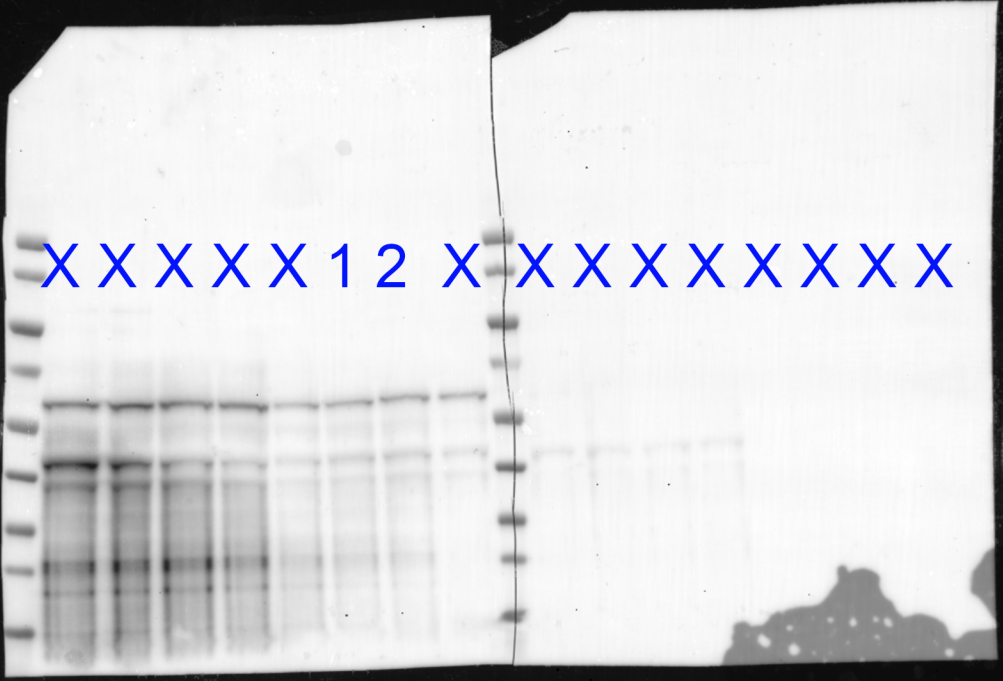

Anti-Chk1

1: Tax1a
2: Tax1b

Figure 6E

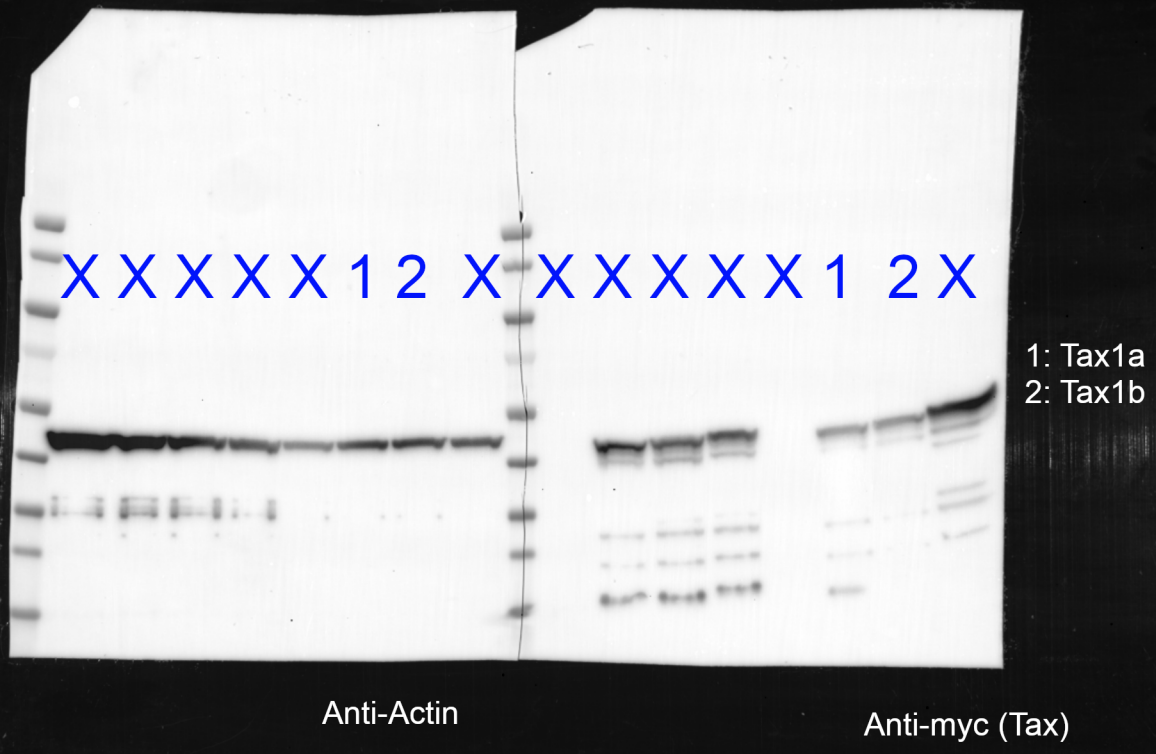

Figure S1B

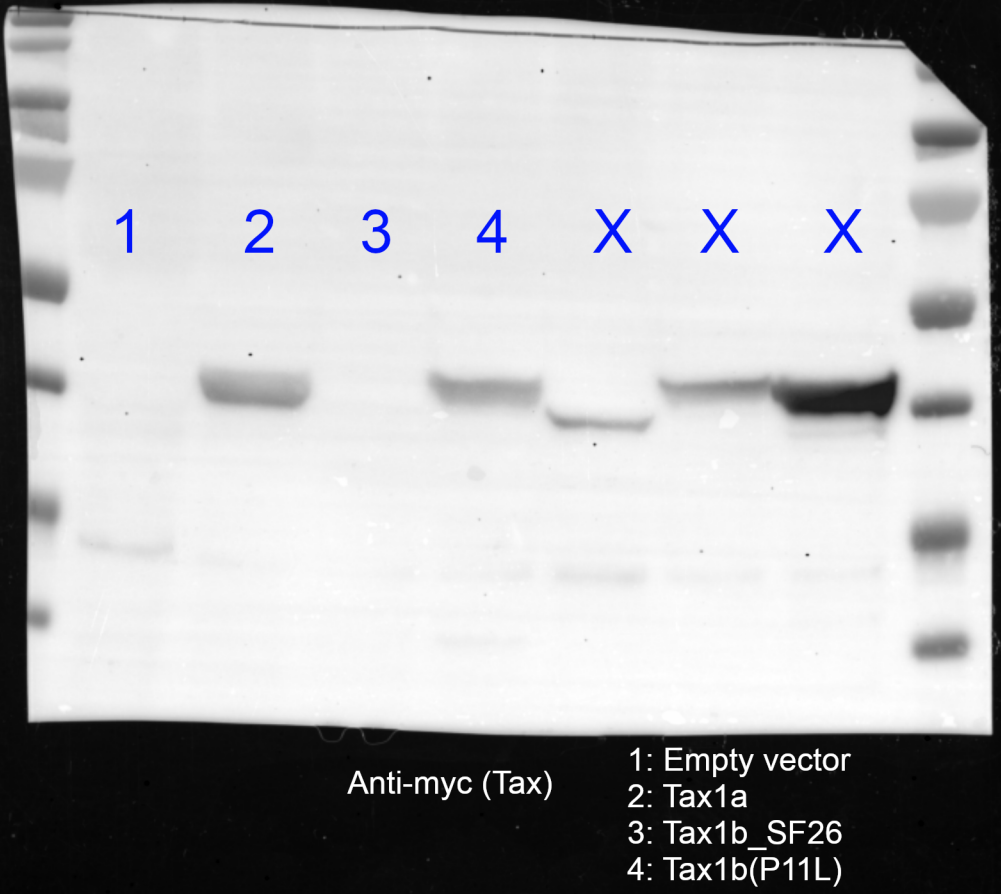
1
2
3
4
X
X
X
Anti-myc (Tax)
1: Empty vector
2: Tax1a
3: Tax1b_SF26
4: Tax1b(P11L)

Figure S1B

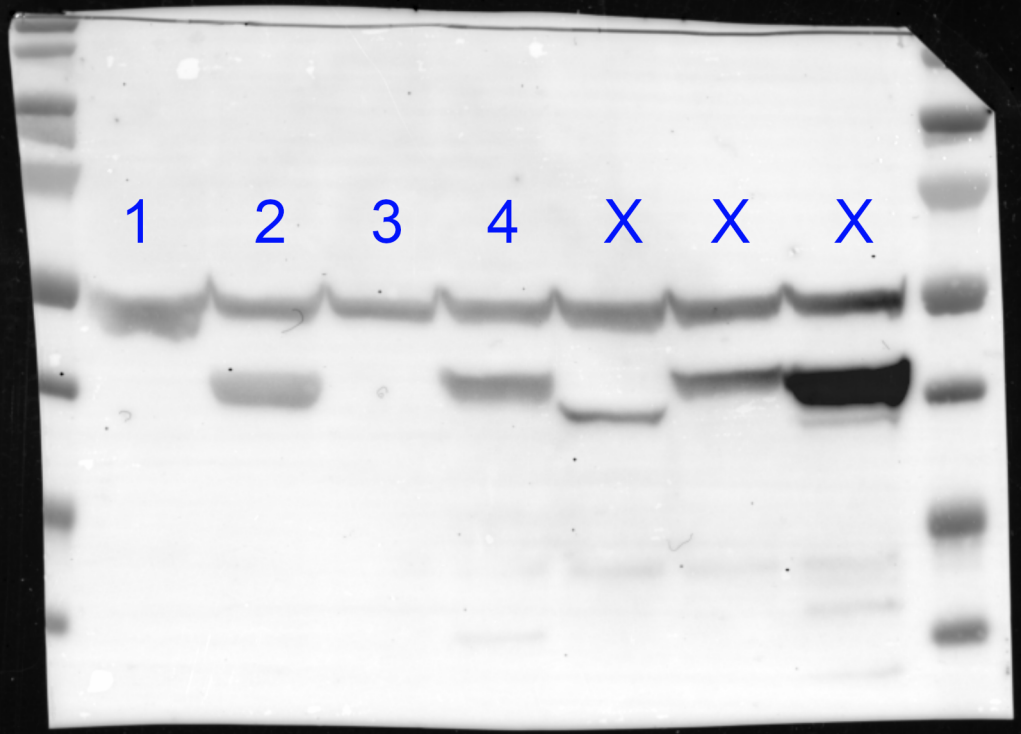

Anti-tubulin (after anti-myc staining)

1: Empty vector
2: Tax1a
3: Tax1b_SF26
4: Tax1b(P11L)

Figure S1E

1: Empty vector
2: Tax1a
3: Tax1b
4: Tax1c-mel
5: Tax1c-aus

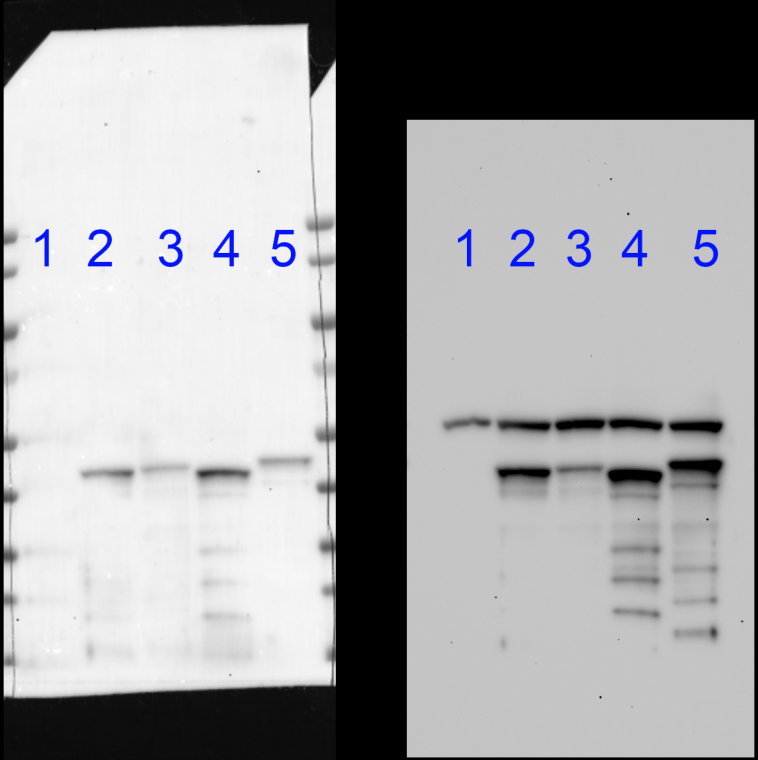

Anti-myc (Tax)

Anti-tubulin (after anti-myc staining)

Figure S3B

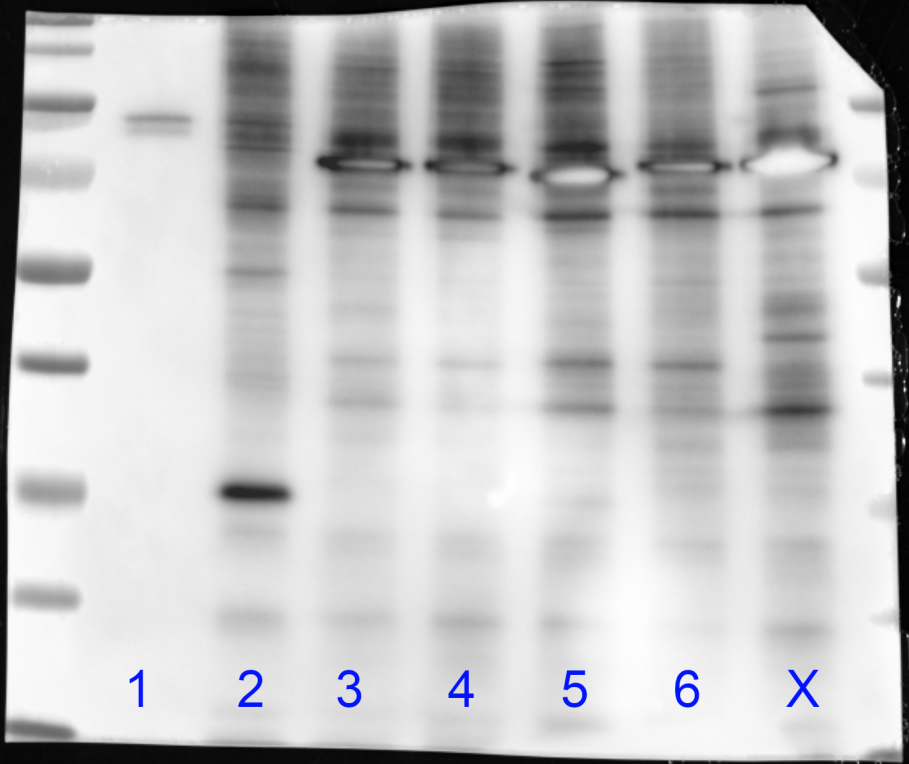

1: Empty vector
2: BirA*
3: BirA*-Tax1a
4: BirA*-Tax1b
5: BirA*-Tax1c-mel
6: BirA*-Tax1c-aus

Streptavidin-HRP
